# A Review of Key Properties of Thermoelectric Composites of Polymers and Inorganic Materials

**DOI:** 10.3390/ma15238672

**Published:** 2022-12-05

**Authors:** Nathan D. Wood, Lisa J. Gillie, David J. Cooke, Marco Molinari

**Affiliations:** Department of Chemical Sciences, School of Applied Sciences, University of Huddersfield, Queensgate, Huddersfield HD1 3DH, UK

**Keywords:** power generation, thermoelectric composite, polymer/inorganic composites, polymer thermoelectric materials, oxide thermoelectric materials, conventional thermoelectric materials

## Abstract

This review focusses on the development of thermoelectric composites made of oxide or conventional inorganic materials, and polymers, with specific emphasis on those containing oxides. Discussion of the current state-of-the-art thermoelectric materials, including the individual constituent materials, i.e., conventional materials, oxides and polymers, is firstly presented to provide the reader with a comparison of the top-performing thermoelectric materials. Then, individual materials used in the inorganic/polymer composites are discussed to provide a comparison of the performance of the composites themselves. Finally, the addition of carbon-based compounds is discussed as a route to improving the thermoelectric performance. For each topic discussed, key thermoelectric properties are tabulated and comparative figures are presented for a wide array of materials.

## 1. Introduction

Current global concerns about energy resources see a move towards sustainable energy generation technologies with improved efficiency and performance, due to a growth in population, a greater demand for electric transportation and machinery, and stringent policies to reduce the environmental impact of anthropogenic activities. To meet such increasing energy demands while maintaining a carbon neutral target, replacing more impactful energy sources with greener solutions remains imperative.

**Figure 1:**
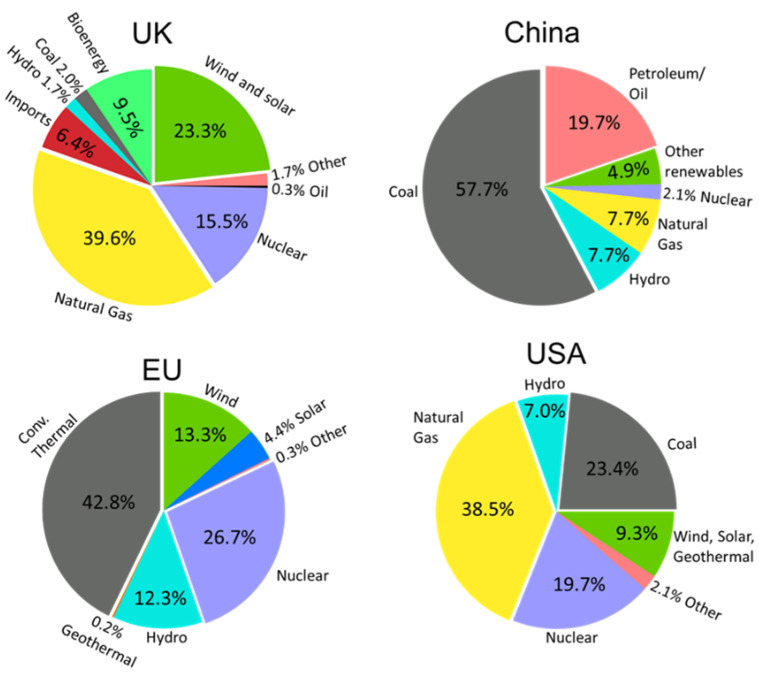
Power generation by fuel type (2019 data) for the UK [2], the EU [3], China [4], and the USA [5]. Conventional thermal includes traditional thermal plants, e.g., coal, oil and gas.

In 2020, the UK total electricity demand approached ~324 TWh with an estimated growth of ~76 TWh predicted by 2040 [1]. Although renewables and nuclear power combined contribute ~50% to the UK total energy generation, and the use of coal is mostly phased out (~2%), other carbon-based fuels, primarily natural gas, still account for ~40%, with plans to reduce this by more than half by 2040 [2]. Although large global economies (e.g., UK [2], EU [3], China [4], and USA [5]) have taken commitments to phase out more impactful energy sources, there is still a clear reliance on such sources (Figure 1). 

For most energy generation technologies, such as thermal power plants and photovoltaics, a substantial portion of energy loss is in the form of heat, leading to lower overall efficiency. In photovoltaics, where the exposure to sunlight is associated with thermal energy, thermoelectric (TE) devices can be implemented to recover the waste heat, converting it directly to electricity [6]. For more conventional methods, which use thermal power, retro-fitting and installation of TE devices would improve the overall power generation capacity [7,8]. 

**Figure 2:**
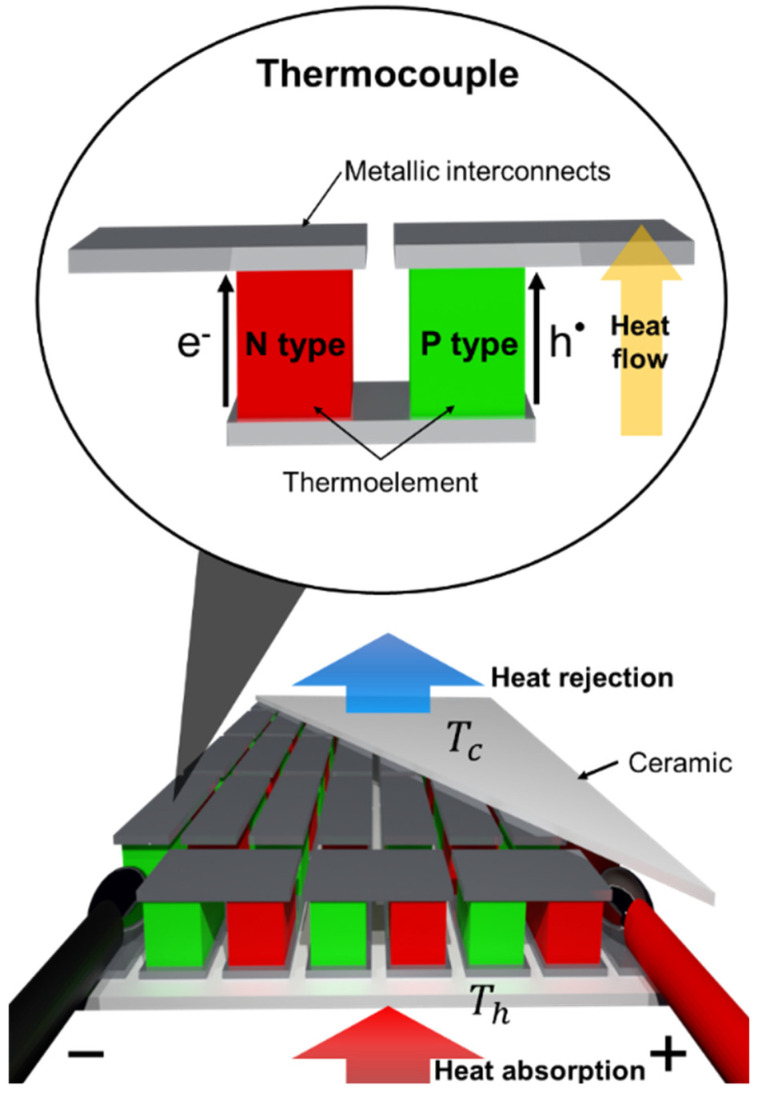
Thermoelectric device illustrating the various components, the direction of heat flow and current for both power generation and cooling. $T_{h}$ and $T_{c}$ are the temperatures of the hot and cold sides, respectively.

A TE device takes the form of a series of thermopiles (Figure 2), i.e., thermocouples wired in series [9,10,11]. A thermocouple consists of two thermoelements connected together in series and thermally in parallel [12]. The two thermoelements are made of n-type and p-type semi-conducting materials, which generate an electrical potential in the presence of a temperature gradient [13,14,15].

To date, the promising use of TE devices is undermined by their low performance and relatively high cost. The overall efficiency of a TE device, $\eta_{max}$, for converting heat into electricity is shown in Equation (1).

(1) $$\eta_{max}=\frac{\Delta T}{T_{h}}\frac{\sqrt{1+Z\times T_{avg}}-1}{\sqrt{1+Z\times T_{avg}}+\frac{T_{h}}{T_{c}}}$$

$\Delta T=T_{h}-T_{c}$ is the difference between the temperatures of the hot $T_{h}$ and cold $T_{c}$ sides, $T_{avg}$ is the average value of ΔT, and Z is the temperature neglected figure of merit. $\eta_{max}$ is presented as a percentage (or decimal), and the efficiency of a typical TE device is around 6–10% [16]. Z is expressed in Equation (2), where S is the Seebeck coefficient, σ is the electrical conductivity, and κ is the thermal conductivity.

(2) $$Z=\;S^{2}\sigma /\kappa$$

To improve the overall efficiency of a TE device, the performance of TE materials is crucial and must be considered on an individual basis. This is measured by the dimensionless figure of merit in Equation (3).

(3) $$ZT=S^{2}\sigma T/\kappa$$

$\kappa =\kappa_{e}+\kappa_{l}$ is the contribution of $\kappa_{e}$ and $\kappa_{l}$, the electrical and lattice thermal conductivities, respectively. $S^{2}\sigma$ is the material’s power factor (PF) described as the total power output in terms of electrical contribution under ideal circumstances where κ is absent. 

The Seebeck coefficient, S, is also known as thermopower and is a measure of the voltage difference across a TE material due to a temperature gradient between its two ends. S depends on the difference in energy between the Fermi energy ($E_{F}$) and the average energy of the charge carrier ($E_{c}$) of the material, as shown in Equation (4).

(4) $$S=\left(\frac{k_{B}}{-q}\right)\left(\frac{E_{c}-E_{F}}{k_{B}T}+\frac{\Delta n}{k_{B}T}\right)=-\frac{E_{c}-E_{F}+\Delta n}{qT}$$

q is the charge of the carrier, T is temperature, $k_{B}$ is the Boltzmann constant, and Δn is the difference in carrier concentration. The resultant sign of S is indicative of the type of conductivity present; n-type and p-type conductivities are associated with a negative and a positive Seebeck coefficient, respectively. The relationship between S and the carrier concentration (n) is further explained by Equation (5):

(5) S=8π2kB23eh2m*T(π3n)23

where $m^{*}$ is the effective mass of the carrier.

The carrier concentration is also related to the electrical conductivity, which represents the ease at which the charge carriers are conducted through the material, Equation (6).

(6) σ=neμ

e is the charge of the carrier and μ is the mobility of the carrier. In TE materials, the ideal n is usually around 10^19^–10^21^ cm^−3^ [17].

The performance of a TE material depends upon an intricate play between the three properties that make up ZT; the Seebeck coefficient, S, the electrical conductivity, σ, and the thermal conductivity, κ. The limitations of TE materials are often centred around the inability to balance all three properties. An ideal TE material would have a large thermopower (S) while minimising ohmic losses, allowing for a high electrical conductivity (σ). There would also be a low thermal conductivity (κ) to maintain the temperature gradient critical to its functionality. It is often the case that any improvement made to one property is marked by the deterioration of another. σ and *S* are related to the charge carrier concentration (n); as n increases so would σ (Equation (6)), which would lead to a decrease in S (Equation (5)). The relationship between S and σ can be further discussed in terms of $m^{*}$ (Equation (5)), which is related to the density of states and increases when there are narrow and flat bands with a high density near the Fermi level. There is also a relationship between $m^{*}$ and the inertial effective mass of carriers. For example, heavier charge carriers move with lower velocities leading to lower mobilities, which in turn lead to a low σ (Equation (6)) [15]. Hence, in cases where $m^{*}$ is large, a high S is achieved, but it comes at the expense of σ.

A change in κ could lead to a noticeable decrease in σ. The Wiedemann–Franz relationship (Equation (7)), describes the dependency of κ on σ, where $\;\kappa_{e}$ is the electron contribution to thermal conductivity and e is the elementary charge of an electron. In instances where there is an increase in n, σ also increases, which leads to a greater $\kappa_{e}$.

(7) $$\frac{\;\kappa_{e}}{\sigma }=\left(\frac{\pi^{2}k_{B}^{2}}{3e^{2}}\right)T$$

Conventional TE materials are crystalline chalcogenides in their bulk form, such as those based on Bi_2_Te_3_ and PbTe, and can offer a respectable *ZT* ≈ 1.1 at room temperature (RT) [18]. However, there are concerns on their toxicity, chemical stability at high temperatures, and low relative abundance of the elements, which render their sustainability questionable [19]. On the other hand, oxide TE materials are economically viable and alternative solutions as they are often cheap and readily available, usually made of abundant elements, and synthesised with simple low-cost processes. The structure of oxides is relatively more versatile in comparison to conventional chalcogenide materials allowing for greater ease in the manipulation of TE properties [20,21,22].

There is mounting evidence that shows competitive performance between oxide and conventional TE materials, with some of the most promising oxides being ZnO (*ZT* = 0.65 at 1247 K) [23], Na_x_CoO_2−δ_ (*ZT* = 1.2 at 880 K) [24], SrTiO_3_ (*ZT* = 0.20 at 1045 K) [25], and CaMnO_3_ (*ZT* = 0.37 at 1000 K) [26], Due to the high thermal stability of oxides [21], the thermal operating window is usually wider than conventional doped materials, e.g., SrTiO_3_ (300–1100 K) [25,27], In_2_O_3_ (300–1100 K) [28], and CaMnO_3_ (300–1000 K) [26]. However, high κ is the major limiting factor of oxide materials, which is more pronounced at lower operating temperatures [20,21,22]. At higher temperatures, κ is usually lower due to phonon–phonon interactions, but unfortunately this is observed at temperatures ≳800 K, which exceeds the majority of waste-heat produced by both industry and transportation (523–723 K) [29]. Indeed, the key difference between oxides and conventional materials is their operating temperature. While oxides operate at high temperatures (>800 K), conventional materials operate ≤800 K with their optimal performance at much lower temperatures (~RT). 

For instance, where there are reasonable PFs (>10^2^ µW m^−1^ K^−2^), it is implied that improvements can be made to the *ZT* of a material, if κ is reduced and both *S* and *σ* are maintained. Material engineering can be attempted by combining different strategies that tend to act on specific TE properties. Nanostructuring aims to introduce grain boundaries and interfaces to reduce thermal conductivity. Nanocompositing aims to mainly reduce thermal conductivity while increasing electrical conductivity by coupling two unalike materials. The adjustment of a TE material’s stoichiometry via the introduction of intrinsic or extrinsic defects is generally named band engineering and mostly aims to increase electrical conductivity and enhance the Seebeck coefficient. Of course, none of these engineering strategies is fully independent with respect to their effect on TE properties. 

For oxides, perhaps the most promising strategies to enhance *ZT* involve the reduction of thermal conductivity. The introduction of large extrinsic dopants, namely “rattling” atoms/dopants, aims to decrease the lattice thermal conductivity ($k_{l}$) by introducing anharmonic motion between dopant atoms and their neighbouring species [30]. This results in a decrease in κ, usually without impacting the electron thermal conductivity ($k_{e}$), and hence maintaining σ, ultimately leading to a higher *ZT*. Nanocompositing, through the creation of composites, is a promising alternative to reduce κ, which has been shown to bring down the operating temperature window [31]. Recently, nanocompositing oxides with carbon allotropes and polymers has been shown to lower $k_{l}$ and hence bring oxides within the operating temperature window of common waste heat (<723 K) with good performance [32,33]. Although many strategies are available to enhance *ZT*, the effectiveness of individual strategies for improvement of TE parameters is limited, and a combination of multiple strategies is generally required. For example, within a TE composite, TE properties are dependent on the constituent materials. While the physics underlying the performance of an individual material may be well understood, there are no general rules on how the material would behave when mixed with other materials to form a composite. Generally, a high weight percentage of the inorganic material constituent in the TE composite allows for the composite to retain similar TE properties of the constituent inorganic material, but there is no general consensus on how the filler loading (i.e., carbon, polymer) may influence the underlying physics. This is a current drawback and hinders the full exploitation of the advantages of TE composites. 

In this review, we cover the composite TE materials made of an inorganic material and a polymer. The term “inorganic (TE) material” covers both oxide and conventional TE materials and is used when discussing both of them. However, when considering specific materials, whether oxides or conventional materials, these terms will be specifically used. As the names suggest, oxides are inorganic materials that contain oxygen, whereas conventional do not. The review is divided into Sections that summarise the inorganic (both conventional and oxide) thermoelectric materials, and their carbon composites (Section 2), polymer(-carbon) thermoelectric materials (Section 3) focusing on those used in inorganic/polymer composites, and finally the inorganic/polymer composites (Section 4). It is important to note that at the beginning of each Section, we refer the reader to complementary reviews. We also note that we cover those inorganic materials used in inorganic/polymer composites, but within Section 2 and Section 3, we also touch on the state-of-the-art materials which report the highest *ZT* values so that a benchmark against the best performing single materials can also be provided. 

## 2. Inorganic Thermoelectric Materials

Here, we provide a brief overview of conventional and oxide TE materials by highlighting the current front-runners (Section 2.1), before delving into the properties of those materials used in inorganic/polymer composites (Section 2.2), and finally discussing the implications of compositing inorganic materials with carbon (Section 2.3). The reader should be aware of promising thermoelectric materials such as clathrates [34] and Zintls [35]; however, as they are not used within inorganic/polymer TE composites, they are not discussed here. 

### 2.1. State-of-the-Art Inorganic TE Materials

While we leave detailed discussions on the potential of oxide and conventional TE materials to several comprehensive reviews [20,21,22,36,37], we briefly revise the current state-of-the-art TE materials (Table 1).

For oxides, the p-type Na_x_CoO_2−δ_ has the highest *ZT* of 1.20 at 800 K. It is clear that oxides have much lower overall performance, with *ZT* values in the range of 0.06–1.2 compared to 0.92–2.80 of conventional TEs. It is also apparent that n-type oxide materials (0.06–0.65) suffer from moderate *ZT* values in comparison to p-type oxide materials (0.29–1.20). Within Table 1, the highest *ZT* are reported for conventional p-type TEs (GeTe)_0.95_(Sb_2_Te_3_)_0.05_ (*ZT* = 2.70 at 720 K) and single-crystal SnSe (*ZT* = 2.80 at 773 K). However, most conventional TEs operate at lower temperatures, ~RT, while oxides reach their optimal performance at higher temperatures, >800 K. A direct comparison cannot thus be made between the two classes of materials, instead here we only highlight the maximum achievable performance of the materials, irrespective of the temperature at which this is achieved. 

When comparing the thermopower of oxides and conventional materials, there is an overall similarity in performance, although some oxides (Sn_0.99_Sb_0.01_O_2_, Ca_0.9_Dy_0.1_MnO_3_ and In_1.88_V_0.12_O_3_) report slightly lower values. For n-type and p-type oxides, the thermopower range is 140–300 µV K^−1^ with an average value of ~217 µV K^−1^. For oxides, the highest *S* values are generally reported for p-type cobaltates, e.g., Bi_2_Sr_3_Co_2_O_y_ (300 µV K^−1^) and Ca_2.97_Sr_0.03_Co_4_O_9_ (270 µV K^−1^), most likely due to the characteristic degeneracy of the 3d orbitals and low-spin state of Co ^3+^ [38]. However, an exception is the n-type perovskite Ba_0.1_Eu_0.9_TiO_3−δ_, which exhibits a large thermopower of −300 µV K^−1^ due to the Eu^2+^ 4f bands located towards the top of the valence band [39], which contribute to increasing *S* [40]. Conventional n-type and p-type materials have a thermopower within the range of *S* = 190–476 µV K^−1^. The highest performing conventional materials have a larger thermopower than all reported oxides, e.g., SnSe (~−476 µV K^−1^) and Sn_0.97_Re_0.03_Se_0.93_Cl_0.02_ (~−430 µV K^−1^).

**Table 1:** Thermoelectric properties of the highest performing inorganic TE materials at a stated temperature (*T*). *σ* is the electrical conductivity, *S* is the Seebeck coefficient, PF is the power factor and κ is the thermal conductivity. All values are reported at the maximum of the figure of merit, *ZT*_max_. ^†^ Calculated from literature values.

Material	*T* (K)	σ	*S* (µV K^−1^)	PF (µW m^−1^ K^−2^)	κ	*ZT*_max_
p-type conventional inorganic materials						
(GeTe)_0.95_(Sb_2_Te_3_)_0.05_ [41]	720	917	217	~4318 ^†^	1.19	2.70
SnSe [42]	923	~96	~342	~1123 ^†^	~0.35	2.60
β-Cu_1.94_Al_0.02_Se [43]	1029	~265	~240	~1526 ^†^	~0.60	2.62
Pb_0.98_Na_0.02_Te-8%SrTe [44]	923	323	294	~2792 ^†^	0.57	2.50
Ge_0.86_Pb_0.1_Bi_0.04_Te [45]	600	365	282	~2903 ^†^	0.49	2.40
Pb_0.92_Mg_0.08_Se_0.2_Te_0.8_ [46]	798	~247	~300	~2223 ^†^	~0.80	2.20
Bi_0.52_Sb_1.48_Te_3_ [47]	310	~643	~230	~3352	~1.35	1.56
Bi_0.88_Ca_0.06_Pb_0.07_CuSeO [48]	873	~163	~228	~862	0.50	1.50
Bi_0.3_Sb_1.7_Te_3_ [49]	378	~859 ^†^	~205	~3610	~1.10	~1.27
n-type conventional inorganic materials						
SnSe [50]	773	~39	~−476	~900	~0.24	2.80
PbTe-4%InSb [51]	773	484	−205	~2034 ^†^	0.25	1.83
Mg_3.175_Mn_0.025_ Sb_1.5_ Bi_0.49_Te_0.01_ [52]	700	~230	~−298	~2042 ^†^	0.55	1.78
Ba_0.08_La_0.05_Yb_0.08_Co_4_Sb_12_ [53]	850	1344	−198	~5269 ^†^	0.40	1.70
Pb_0.93_Sb_0.05_S_0.5_Se_0.5_ [54]	900	421	−188	~1488 ^†^	0.80	1.65
Sn_0.97_Re_0.03_Se_0.93_Cl_0.02_ [55]	798	~32	~−430	~578	~0.32	1.50
Bi_1.8_Sb_0.2_Te_2.7_Se_0.3_+15%Te [56]	425	819	−198	~3211 ^†^	0.38	1.40
Bi_2.0_Te_2.7_Se_0.3_ [57]	300	270	−200	~1080 ^†^	0.22	1.47
Ba_8_Ga_15.8_ Cu_0.033_Sn_30.17_ [58]	550	187.2	−307	~1764 ^†^	0.70	1.38
(Hf_0.5_Zr_0.5_)_0.7_Ti_0.3_NiSn_0.998_Sb_0.002_ [59]	873	~928	~190	~3740	~3.10	0.92
p-type oxide inorganic materials						
Na_x_CoO_2−δ_ [24]	800	1923	200	7690	5.10	1.20
Bi_0.94_Pb_0.06_CuSeO [60]	823	135	221	~659 ^†^	0.60	1.14
Bi_2_Sr_3_Co_2_O_y_ [61]	973	-	300	-	2.00	1.10
Ca_2.87_Ag_0.05_Lu_0.16_Co_4_O_9+δ_ [62]	1118	-	~232	-	~1.40	~0.60
Ca_2.97_Sr_0.03_Co_4_O_9_ [63]	1073	~154	270	1200	4.40	0.29
n-type oxide inorganic materials						
Zn_0.96_Al_0.02_Ga_0.02_O [23]	1247	~570	~−250	~2309	~4.82	0.65
Ca_0.97_Bi_0.03_MnCu_0.04_O_3−δ_ [64]	1073	120.7	~−214	~553 ^†^	1.50	0.44
In_1.88_V_0.12_O_3_ [65]	973	~386	~−141	~777	~1.78	0.42
La_0.08_Sr_0.92_TiO_3_ [25]	1045	~210	~−232	~1130 ^†^	~3.05	0.37
TiO_1.76_ [66]	973	350	~−148	~767 ^†^	2.10	0.35
Ba_0.1_Eu_0.9_TiO_3−δ_ [39]	1123	~63	−300	~567 ^†^	2.70	0.24
Ca_0.9_Dy_0.1_MnO_3_ [26]	1000	~150	~−155	~359	~1.85	0.20
Sn_0.99_Sb_0.01_O_2_ [67]	1073	183	−159	460	7.94	0.06

For the electrical conductivity, σ, the range for oxides is generally reported between 63–210 S cm^−1^, but the reported values for Na_x_CoO_2−δ_ (single-crystal, 1923 S cm^−1^), Zn_0.97_Al_0.02_Ga_0.02_O (~570 S cm^−1^), In_1.88_V_0.12_O_3_ (385.5 S cm^−1^) and TiO_1.76_ (350 S cm^−1^), show that they outperform some of the best conventional materials. Indeed, the highest reported σ for the oxide Na_x_CoO_2−δ_ (1923 S cm^−1^) far exceeds that of the highest σ for the conventional TE Ba_0.08_La_0.05_Yb_0.08_Co_4_Sb_12_ (1344 S cm^−1^). For both conventional and oxide TEs, smaller σ values may be attributed to a low carrier concentration and/or the nature of the charge carriers [68]. 

When comparing TEs in terms of power factor, PF ($S^{2}\sigma$), which represents the material’s performance independently of thermal conductivity, oxides report higher values in the range of 350–7690 µW m^−1^ K^−2^, while conventional TEs report PF in the range of 570–5269 µW m^−1^ K^−2^. For some of the most promising oxides, e.g., Zn_0.97A_l_0.02_Ga_0.02_O (~2309 µW m^−1^ K^−2^ at 1247 K) and Ca_2.97_Sr_0.03_Co_4_O_9_ (1200 µW m^−1^ K^−2^ at 1073 K), larger PFs are observed at high temperatures. It is seen that the highest PFs in oxides are reported almost exclusively for the p-type layered and misfit cobaltates, with the exception of the n-type zinc oxide, Zn_0.97_Al_0.02_Ga_0.02_O. It is also apparent that some of the perovskite-type structures perform particularly well, e.g., La_0.08_Sr_0.92_TiO_3_ (~1130 µW m^−1^ K^−2^ at 1045 K). The oxide with the highest PF is Na_x_CoO_2−δ_ (7690 µW m^−1^ K^−2^), which is reported at a much higher temperature (800 K) compared to the several high-performing conventional TEs (e.g., (GeTe)_0.95_(Sb_2_Te_3_)_0.05_ and (Hf_0.5_Zr_0.5_)_0.7_Ti_0.3_NiSn_0.998_Sb_0.002_). It is also the case that the highest PF reported for conventional materials is the cobalt containing Ba_0.08_La_0.05_Yb_0.08_Co_4_Sb_12_, where PF = ~5269 µW m^−1^ K^−2^ at 850 K. 

Thermal conductivity, κ, varies substantially between conventional and oxide materials, with oxides displaying a consistently higher κ. Oxides exhibit a κ in the range of 0.60–7.94 W m^−1^ K^−1^ at 800–1247 K, while conventional TEs are in the range 0.22–3.10 W m^−1^ K^−1^ at 310–1029 K. The disparity between values of κ is perhaps attributed to the fact that many of the conventional materials are layered structures (e.g., Bi_2_Te_3_) or have bonding which is less conducive for phonon-transport, resulting in a low $\;\kappa_{l}$, e.g., anharmonicity, bond heterogeneity, emphanitic anharmonic behaviour and intrinsic rattling modes [69]. It is the case that many n-type oxides are not layered and hence express a greater phonon propagation throughout the structure leading to a greater κ. There are also layered oxide materials belonging to the family of cobaltates [70,71], which have an intrinsically low κ due to the mismatch between the layers, layers’ stacking and layers’ composition [72,73].

### 2.2. Inorganic TE Materials Used in Inorganic/Polymer Composites

**Figure 3:**
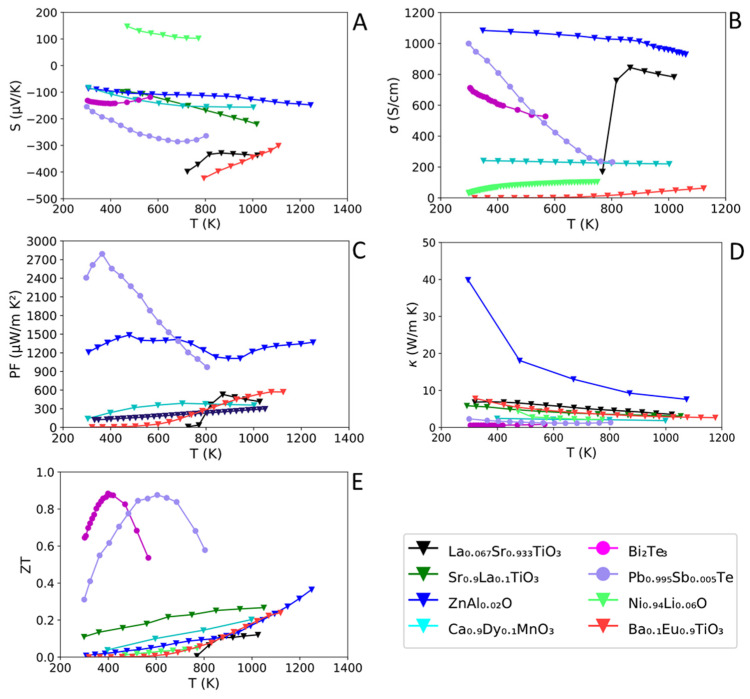
Comparison *S* (**A**), *σ* (**B**), PF (**C**), κ (**D**) and *ZT* (**E**) for a number of inorganic TE materials from Table 2 as a function of temperature (K). La_0.067_Sr_0.933_TiO_3_ [31], Sr_0.9_La_0.1_TiO_3_ [77], Zn_0.998_Al_0.02_O [23], Ca_0.9_Dy_0.1_MnO_3_ [26], Bi_2_Te_3_ [75], Pb_0.995_Sb_0.005_Te [74], Ni_0.94_Li_0.06_O [79], and Ba_0.1_Eu_0.9_TiO_3–δ_ [39]. CaMnO_3_ has not yet been used within composite materials but is a popular TE candidate.

Here, the performance of oxides and conventional TE materials typically used within inorganic/polymer composites are discussed, irrespective of their n-type and p-type behaviour. Although we recognise that some of these materials could show a higher *ZT* with nanostructuring/band engineering, when they are used in composites, the materials are usually not fully optimised. Furthermore, for many of the inorganic/polymer composites discussed in Section 4, the chemical composition of the inorganic TE materials is not reported, so here we cover typical compositions for these TE materials. Table 2 summarises TE properties at *ZT*_max_ for conventional and oxide TE materials used within inorganic/polymer composites. Figure 3 shows *ZT*, PF, *σ*, *S* and κ for the inorganic TE materials over their respective temperature range of applications. This Section is fundamental for the understanding of the effect of polymers on the properties of their corresponding inorganic/polymer composites in Section 4. 

**Table 2:** Thermoelectric properties of various inorganic materials typically used in inorganic/polymer composites at a stated temperature (*T*). * Data reported at the temperature in brackets. *σ* is the electrical conductivity, *S* is the Seebeck coefficient, PF is the power factor and κ is the thermal conductivity. All values are reported at the maximum of the figure of merit, *ZT*_max_. ^†^ Calculated from literature values.

Material	*T* (K)	σ	*S* (µV K^−1^)	PF (µW m^−1^ K^−2^)	κ	*ZT*_max_
n-type conventional inorganic materials						
Pb_0.995_Sb_0.005_Te [74]	600	~210 ^†^	~−280	~1692	~1.15	~0.87
Pb_0.995_Sb_0.005_Te [74]	300	1000	~−157	~2410	~2.31	~0.32
Bi_2_Te_3_ [75]	400	~608	~−143	~−1249 ^†^	~0.59	0.88
Bi_2_Te_3_ [76]	325	~483	~−141	~960	~1.29	~0.25
n-type oxide inorganic materials						
La_0.1_Sr_0.9_TiO_3_ [77]	1050	~80 (950 K) *	~−289	~668 ^†^	~3.10	~0.27
La_0.067_Sr_0.933_TiO_3_ [31]	1023	~35 ^†^	~−340	~416	~3.55	~0.12
Ba_0.1_Eu_0.9_TiO_3–δ_ [39]	1123	63	−300	~570	~2.70	~0.24
Zn_0.998_Al_0.02_O [23]	1247	~630	~−150	~1370	~7.60 (1073 K) *	~0.37
p-type oxide inorganic materials						
Ca_2.93_Sr_0.07_Co_4_O_9_ [78]	1047	~62	~219	~298	-	-
Ni_0.94_Li_0.06_O [79]	770	~104	~103	~110 ^†^	~2.19	~0.05

The most impressive *S* values are reserved for oxides (Table 2), 100–340 µV K^−1^, while conventional TEs are within the 140–280 µV K^−1^ range. The oxides La_0.1_Sr_0.9_TiO_3_ (~−340 µV K^−1^, 1023 K) and Ba_0.1_Eu_0.9_TiO_3–δ_ (~−300 µV K^−1^, 1123 K) are relatively impressive, outperforming the conventional material Pb_0.995_Sb_0.005_Te (~−280 µV K^−1^ at 600 K), which can be classified as a competitor due to its performance in proximity of the operating temperature of oxides (>800 K). *S* has many dependences on both charge carrier concentration (n) and effective mass of the carrier ($m^{*}$) as shown in Equations (5) and (6), respectively. Within many oxides, n is usually small, whilst $m^{*}$ is often large, which explains why oxides have a much larger *S* than conventional materials [22,80]. Temperature effects on *S* are highly dependent upon the nature of the structure of the TE material. Indeed, *S* (Equations (4) and (5)) is dependent upon the parameters $E_{F}$, $E_{c}$, μ, n and $m^{*}$, which are distinctive for each individual structure.

Figure 3A shows the effect of temperature on *S* for inorganic TEs. For some oxide and conventional TEs, there is very little change in *S* with increasing temperature, e.g., Bi_2_Te_3_ (~10%, 300–565 K), Ba_0.1_Eu_0.9_TiO_3–δ_ (~34%, 300–793 K) and Ni_0.94_Li_0.06_O (~−36.21%, 470–770 K), whereas for others there is a more significant change, e.g., Zn_0.998_Al_0.02_O (~−54%, 305–1245 K), and Pb_0.995_Sb_0.005_Te (~−52%, 298–803 K). 

The electrical conductivity of oxides shown in Table 2 is generally in the range of 35–104 S cm^−1^ except for Zn_0.998_Al_0.02_O (~630 S cm^−1^), which like its state-of-the-art counterpart Zn_0.97_Al_0.02_Ga_0.02_O (Table 1), has a high σ (~570 S cm^−1^). Oxides show poor electrical conductivity compared to conventional materials, which display σ in the range of 210–1000 S cm^−1^, with the highest value of 1000 S cm^−1^ for Pb_0.995_Sb_0.005_Te at 300 K, followed by 608 S cm^−1^ for nanostructured Bi_2_Te_3_. Figure 3B shows the effect of temperature on σ for both oxides and conventional TEs. The majority of oxides show a large operating temperature range of 300–1000 K. The electrical conductivity has very different trends due to the different nature of the structures: it increases with temperature for Ba_0.1_Eu_0.9_TiO_3–δ_ and Ni_0.94_Li_0.06_O, it remains fairly constant in Ca_2.93_Sr_0.08_Co_4_O_9_, and decreases with temperature for Zn_0.998_Al_0.02_O. Perovskite La_0.067_Sr_0.993_TiO_3_ shows a more complicated relationship where a reasonable conductivity (~760 S cm^−1^) is shown at T > 800 K, after a sharp rise in σ from just below 800 K (~170 S cm^−1^). 

Conventional TEs have a maximum σ at ~RT and display a sharp reduction in σ with increasing temperature. The different behaviour of σ with temperature between conventional and oxide materials is attributed to the interaction of electrons with phonons explained by the Wiedemann–Franz law (Equation (7)). Reduction of σ at high temperatures for both oxides and conventional materials is also attributed to a higher energy of the phonon-electron interactions, which scatter electrons via the Umklapp scattering [81]. When a material is subjected to this mechanism, a characteristic visible decrease in κ in line with temperature is usually observed.

Figure 3C shows the effect of temperature on PF. Conventional materials (Figure 3C, Table 2) have higher PFs (>950 µW m^−1^ K^−2^) than oxides, which usually sit within 100–600 µW m^−1^ K^−2^. However, the oxide Zn_0.998_Al_0.02_O (~1370 µW m^−1^ K^−2^ at 1247 K) shows a comparable performance to conventional materials and indeed exceeds the performance of Bi_2_Te_3_ (~934 µW m^−1^ K^−2^ at 325 K) irrespective of the operating temperature. As discussed previously (Section 2.1), *S* and σ must be considered here as PF is reliant upon both these parameters, and hence temperature effects on PF vary from material to material. 

Oxides used in inorganic/polymer composites (Figure 3E, Table 2) express lower *ZT* (0.05–0.37) than conventional TE materials (0.31–0.88). Nevertheless, a few promising oxides La_0.1_Sr_0.9_TiO_3_ (~0.27 at 1050 K), Ba_0.1_Eu_0.9_TiO_3–δ_ (~0.24 at 1120 K) and Zn_0.998_Al_0.02_O (~0.37 at 1247 K) display *ZT*_max_ values similar to the lowest reported conventional material Pb_0.995_Sb_0.005_Te (0.32 at 300 K). This suggests that a number of oxides show a similar performance compared to conventional TEs, and hence could yield similar or better performance at their individual optimal temperature ranges. 

As the PFs of oxides are typically ⪆200 µW m^−1^ K^−2^, their lower *ZT* is mainly resultant from the larger κ, which is usually in the range of 2–8 W m^−1^ K^−1^ compared with conventional materials (0.5–2.4 W m^−1^ K^−1^). Astonishingly, at RT, Na_x_CoO_2−δ_ has a low *ZT* = 0.036, which is attributed to a large κ of 16.5 W m^−1^ K^−1^ due to its layered structure [24,82].

For most oxides, *ZT* values increase over the temperature range, Figure 3E, indicating the possibility of a wide optimal operating temperature window. For conventional materials, there is a steep “normal” distribution over a narrow range of temperature (~300–800 K) indicating a limited optimal operating window, if consistent values of *ZT* need maintaining. For numerous oxides shown in Figure 3C, the PF is rather steady and consistent over their reported temperature ranges, suggesting that oxides may be suited (if engineered correctly) for use over a wide temperature range (300–1100 K). However, as PF disregards κ, to establish a wide operating temperature window, the inherently high κ of the oxides (particularly near RT) needs to be reduced and controlled. This would give oxides an advantage over conventional materials as the latter are chemically unstable at high temperatures (>800 K). Currently, despite oxides being better suited in theory for a wide temperature range, the more impressive *ZT* values are limited to high temperatures (>800 K) with very low values reported under ambient conditions. 

### 2.3. Inorganic/Carbon TE Composites 

The addition of carbon allotropes to inorganic TE materials is a relatively new area of research with promising results obtained thus far. The manufacturing of carbon composites has been shown to broaden the operating temperature range and provide a lower temperature at which maximum *ZT* is achieved, allowing for possible lower-temperature applications (RT) [31]. We make use of specific examples to justify the manufacturing of inorganic/carbon composites, and explain how the addition of carbon fillers to polymer/inorganic composites may be beneficial. We leave their detailed discussion to a number of reviews [37,83,84,85,86,87]. As we are only considering carbon as an additive, for further information on the electronic and thermal properties of carbon materials including graphene, we refer the reader to several reviews [88,89,90]. Furthermore, chirality of carbon nanotubes (CNTs) and their wide variety of complex structures can have great influence on the thermal properties [91,92,93,94].

Within this Section, examples of inorganic/carbon composites are discussed, listing some high performing composites and the rationale behind their applicability is given. A comparison to oxides and conventional TEs is made. The creation of composites usually involves the addition of organics and/or more recently polymers to a doped inorganic material, which is either in bulk or nanoparticle forms. Table 3 summarises the TE parameters of high performing conventional and oxide carbon composite TEs at *ZT*_max_.

The creation of interfaces has been explored in La and Nb-doped SrTiO_3_ [31,32,95]. Graphene was incorporated into their polycrystalline microstructure via a nanoparticle-like grain-boundary engineering approach [31,95]. These composite nanostructures greatly influenced the TE properties. The inclusion of 0.23 vol. % (1 wt. %) of graphene to La_0.067_Sr_0.933_TiO_3_ generated a σ = ~8200 S cm^−1^, which is greater than that of the inorganic single crystal (σ = ~7500 S cm^−1^), leading to a PF = ~2400 µW m^−1^ K^−2^ at ~312 K, which is marginally larger than the inorganic single crystal (PF = ~2370 µW m^−1^ K^−2^ at ~348 K) [31,95]. The formation of a multi-phase structure with nano-sized grains was consistent with varied loadings of graphene, which led to a decrease in *κ*, an increase in *σ*, while moderate *S* values were maintained [31]. With lower graphene loadings, Gr (0.6 wt. %)/La_0.067_Sr_0.933_TiO_3_, a high PF = ~2500 μW m^−1^ K^−2^ was achieved, leading to *ZT* = 0.42 at RT, which is an approximately 280% increase compared to inorganic La_0.067_Sr_0.9_TiO_3_, and slightly higher than that of Gr (1 wt. %)/La_0.067_Sr_0.9_TiO_3_. This could be attributed in part to the large reduction in κ, which was lowered to ~25% of that of pure La_0.067_Sr_0.9_TiO_3_ at 315 K due to the scattering of phonons at the interfaces. The differences in TE parameters between varied loadings of graphene suggest that there is an optimal loading window for the addition of graphene, too much or too little can hinder performance. The TE parameters of the aforementioned composites are comparable at RT to those typically reported for conventional materials (Table 2, Figure 3) [31]. For undoped SrTiO_3_, the addition of 0.11 vol. % graphene, above 700 K was also found to exhibit single-crystal-like carrier mobility, yielding to a *S* = −369.38 µV K^−1^ and a σ = ~5000 S cm^−1^. [95] This suggests that the role of graphene for increasing the electrical TE parameters can be independent of the doping approach, and hence may be an independent and useful strategy to improve performance of inorganic/polymer composites. 

The operating window of the oxide/carbon composite Gr/La_0.067_Sr_0.9_TiO_3_ (from RT to ~400 K) has opened up to lower temperatures, in line with conventional TEs such as Bi_2_Te_3_ and PbTe. This change was also observed upon the addition of reduced graphene oxide (RGO) to undoped SrTiO_3_ [96]. The addition of RGO also leads to single-crystal-like electron mobility. It has been theorised that the improvement of *σ* is due to mitigation of electrical impedance induced by trapped electrons at the grain boundaries. The addition of RGO to SrTiO_3_ leads to formation of strontium ($V_{Sr}^{\prime \prime }$) and oxygen ($V_{O}^{••})$ vacancies at the RGO-SrTiO_3_ interface which in turn influences the band structure, lowering the “double Schottky energy barrier” and hence improving *σ* [96]. As $V_{O}^{••}$ are electron donors, they also contribute to the enhancement of *σ* [97].

**Table 3:** Thermoelectric properties of inorganic/carbon thermoelectric composite materials. *σ* is electrical conductivity, *T* is temperature, *S* is the Seebeck coefficient, PF is the power factor and κ is the thermal conductivity. All values are reported at *ZT*_max_, figure of merit at its maxima. G = graphite, Gr = graphene. ^†^ Calculated from literature values.

Carbon Composite	*T* (K)	σ	*S* (µV K^−1^)	PF (µW m^−1^ K^−2^)	κ	*ZT*_max_
n-type inorganic/carbon composites						
G (0.5 wt. %)/SrTi_0.85_Nb_0.15_O_3_ [98]	1047	~1193	~−187.00	~4237	~3.13	1.42
G (1.0 wt. %)/La_0.07_Sr_0.93_Ti_0.93_Nb_0.07_O_3_ [99]	1023	~572	~−193	~2147	~3.14	~0.68
Gr (1.5 wt. %)/Bi_2_Te_3_ [100]	495	~734	~−124	~1130 ^†^	~1.04	~0.55
Gr (0.6 wt. %)/La_0.067_Sr_0.9_TiO_3_ [31]	315	~1780	~−117	~2500	~1.80	0.42
Gr (3.0 wt. %)/PbTe [101]	300	~234	~−200	~936	~0.93	~0.30
SWCNT (0.5 wt. %) (0.7–1.4 nm, 0.5–2 µm)/Bi_2_Te_3_ [102]	300	~200	−231.2	~1069	1.20	~0.27
Gr (1.0 wt. %)/Sr_0.8_La_0.067_Ti_0.8_Nb_0.2_O_3-*δ*_ [32]	1000	~238	~−173	-	~2.8	0.25
CNT (10 wt. %)/TiO_2_ [103]	300	0.71	−552.8	21.5	2.43	3.04 × 10^−3^
p-type inorganic/carbon composites						
CNT (0.05 wt. %)/PbTe [104]	325	~123	~308	~1163	~0.88	~0.42
Graphene/Cement/Fe_2_O_3_ [105]	343	~8	107	9.5	~0.66	5 × 10^−3^
Graphene/Cement/ZnO [105]	343	~14	141	28	~1.02	1.01 × 10^−2^
Graphene/Cement/MnO_2_ [105]	338	~6	99.5	5.5	~0.95	2 × 10^−3^

Within Table 3, the TE composite with the highest performance in terms of *ZT* is G (0.5 wt. %)/SrTi_0.85_Nb_0.15_O_3_, *ZT* = 1.42 at 1047 K, which is a considerable increase compared to the highest performing doped SrTiO_3_, e.g., La_0.08_Sr_0.92_TiO_3_ (Table 1, *ZT* = 0.37, 1045 K) and Gr (1.0 wt. %)/Sr_0.8_La_0.067_Ti_0.8_Nb_0.2_O_3-*δ*_ (Table 3, *ZT* = 0.25, 1000 K). This is perhaps attributed to the large *σ* = 1193 S cm^−1^ and a reasonable *S* = −187 µV K^−1^, as in this case κ = 3.13 W m^−1^ K^−1^, which is similar to that reported in oxide inorganic, La_0.08_Sr_0.92_TiO_3_ (Table 1, κ = 3.05 W m^−1^ K^−1^, 1045 K), suggesting that the high *ZT* value cannot be attributed to the slight reduction in κ. For the addition of graphene in La_0.067_Sr_0.9_TiO_3_ (Table 3), κ decreases compared to La_0.067_Sr_0.9_TiO_3_ (~3.55 at 1050 K, Table 2), which is particularly apparent at low temperatures (<600 K).

For conventional/carbon composite, graphene (1.5 wt. %)/Bi_2_Te_3_, *ZT* = 0.55 at 495 K is shown to be an increase from that of Bi_2_Te_3_ (Table 2, *ZT* = 0.25 at 325 K). This may be attributed to the slight decrease in κ = 0.25 W m^−1^ K^−1^ due to the graphene–Bi_2_Te_3_ interface and the increase in σ = ~90 S cm^−1^ brought on by the intrinsically high σ of graphene. However, the increase in σ and decrease in κ are also marked by a slight decrease in the magnitude of *S* = ~19 µV K^−1^. The performance of this composite is also closely followed by Graphene (0.6 wt. %)/La_0.067_Sr_0.9_TiO_3_ and CNT (0.05 wt. %)/PbTe. 

As discussed within this Section, the creation of composites with carbon can lead to beneficial changes to key thermoelectric parameters. Indeed, although at the expense of *S*, κ is often reduced, and *σ* is increased. Similar results are expected for the manufacturing of inorganic/polymer composites. 

## 3. Polymer Thermoelectric Materials

Polymer TEs obey the same fundamental principles as inorganic TEs (Equations (1)–(7)). However, thermal and electronic transport may require more complex considerations; we refer the reader to the review of Xu et al. [106]. Most polymers in their undoped form are electrical insulators with innately low electrical conductivity (10^−6^–10^−11^ S cm^−1^), and usually have a relatively wide bandgap of 2–3 eV [107]. Chemical doping is often employed to enhance σ by increasing the number of charge carriers and their concentration (n). Doping usually takes place by either reducing or oxidising the polymer [108]. The increase in σ leads to organic semi-conductors with a more pronounced TE performance. Although the relationship between σ and n in Equation (6) may not be entirely suitable for the description of the electrical conductivity in doped polymers due to their disordered nature and complicated band structures, it still used throughout literature discussing TE polymers and serves as an effective guideline description [109,110]. Other charge transport models have been tabulated in the work of Gregory et al. [111], whereas a discussion is provided by Kaiser [112]. 

Whilst the n-type polymers discussed in this Section cannot be easily split in to general classes, the p-type polymers can be split into two classes: those containing sulphur, i.e., polythiophenes (PTh, e.g., PEDOT:PSS and P3HT) and those containing nitrogen. The latter can be further divided into two categories: nitrogen within the aromatic system (NAr, e.g., PPy), and nitrogen outside the aromatic system (N, e.g., PANI). Here, we begin by introducing the current front-runners in polymer TE materials (Section 3.1) to highlight the current maximum TE performance that polymers can offer. We then cover polymers used within both oxide and conventional inorganic/polymer TE composites known so far (Section 3.2). Finally, we discuss polymer/carbon TE composites (Section 3.3), and where feasible, we have compared different classes of materials, in terms of highest performance achievable.

### 3.1. State-of-the-Art Polymer TE Materials

Table 4 summarises the TE properties at *ZT*_max_ for the highest performing polymer thermoelectrics within the current literature. We leave detailed discussion of the potential of polymers for TE applications to several comprehensive reviews [113,114,115].

While all the TE polymers in Table 4 operate at around RT, those that show exceptional performance are found to be p-type. The n-type polymers exhibit an overall poor σ (~4–23 S cm^−1^). When σ is considered, p-type TE polymers fall into the range of 220–1600 S cm^−1^ (Table 4), which demonstrates that polymers perform as well as the many of the front-running conventional (39–1344 S cm^−1^, Table 1) and oxide (63–1923 S cm^−1^, Table 1) TEs. The highest σ, 1600 S cm^−1^, is reported for p-type acid-base treated PEDOT:PSS/EMIM-DCA, which exceeds all front-running conventional (Ba_0.08_La_0.05_Yb_0.08_Co_4_Sb_12_, 1344 S cm^−1^) TEs. The polymer PEDOT:PSS/EMIM-DCA also exceeds the highest reported σ for typical inorganic TEs, which are currently used in inorganic/polymer TE composites (i.e., Zn_0.998_Al_0.02_O, ~630 S cm^−1^, Table 2), making it a promising candidate for compositing. Such impressive electrical conductivities of TE polymers can be attributed to the nature of covalent bonding and the ability for bond conjugation, which is influenced and modulated by the functionality of the polymer and chemical doping. The manipulation of the electronic states involved in bond conjugation leads to the alteration of both carrier concentration (n) and mobility of the carrier (μ) (Equation (6)). [125]. When comparing σ between polymer’ classes for p-type polymers, there is a clear disparity between N class (e.g., PANI, 220 S cm^−1^), which express much lower σ, and even the lowest performing PTh polymer, P3HT (320 S cm^−1^).

p-type polymers outperform n-type polymers in terms of σ, with σ in the range of 190–380 µV K^−1^. This makes them competitive compared with some of the top performing inorganic materials, e.g., Pb_0.92_Mg_0.08_Se_0.2_Te_0.8_ (300 µV K^−1^, Table 1). The magnitude for *S* is fundamentally low for p-type polymers, in the range of 20–117 µV K^−1^, which is overshadowed by both the highest performing oxide (141–300 µV K^−1^) and conventional (190–476 µV K^−1^) TEs (Table 1). However, PP-PEDOT:Tos (~117 µV K^−1^) shows great promise, with a magnitude of *S* similar to a number of the highest performing inorganic TEs. It is important to remember that both *S* and σ are interdependent (Equations (4)–(6)), and while high σ values are reported for p-type polymers, these come at the expense of *S*. This can be explained by the relationship between σ, carrier concentration (n), and mobility of the carrier (μ). A high σ is indicative of both large n and μ. Since a large μ relies on high velocity of the charge carrier, which is related to $m^{*}$, it means that all the shared parameters between *S* and σ tend to favour a high σ leading to a low *S*. Conversely, the use of various dopants may alter the effective mass of the carrier $m^{*}$ (Equation (5)) and lead to an improvement in *S*. When we consider the different polymer classes, the N polymer, PANI (~20 µV K^−1^) exhibits the lowest *S* value, which in performance is similar to the lowest reported PTh polymer, PBTTT/FTS (33 µV K^−1^).

Many papers investigating TE properties of polymers report only values for PF instead of *ZT*. The PF of p-type polymers is in the range of 11–1270 µW m^−1^ K^−2^, while the ranges of PFs for conventional and oxide TEs are 578–5269 µW m^−1^ K^−2^ and 359–7690 µW m^−1^ K^−2^, respectively (Table 1 and Table 2, Figure 3). The n-type polymers have a much lower PF range of 16–80 µW m^−1^ K^−2^ compared to the p-type polymers (Table 4) that are on the whole comparable to some typical oxide TEs, e.g., La_0.067_Sr_0.993_TiO_3_ (~416 µW m^−1^ K^−2^) and Ca_2.93_Sr_0.08_Co_4_O_9_ (~298 µW m^−1^ K^−2^). It is clear that most polymers do not compete with conventional TEs at RT, e.g., Pb_0.995_Sb_0.005_Te (~2410 µW m^−1^ K^−2^ at 325 K), apart from the front-runners acid-base treated PEDOT:PSS/EMIM-DCA (754 µW m^−1^ K^−2^) and PP-PEDOT:Tos (1270 µW m^−1^ K^−2^ at 300 K). Additionally, when comparing between polymer classes, the N polymer, PANI (~11 µW m^−1^ K^−2^) has the lowest PF of the p-type polymers, far less than the lowest reported for the PTh class, e.g., P3HT (~46 µW m^−1^ K^−2^). 

Polymers show inherently low thermal conductivities (0.23–0.52 W m^−1^ K^−1^, Table 4), compared to conventional (0.24–3.10 W m^−1^ K^−1^) and oxide TEs (0.60–7.94 W m^−1^ K^−1^) over their operating ranges (Table 1 and Table 2, Figure 3). This low κ is explained by the propagation of phonon modes, and the linear nature in which they tend to travel through bonded sets of atoms. Within polymers the disordered nature of their structure leads to scattering and hence low propagation of phonon modes [126]. When comparing the κ of polymers with oxide (Na_x_CoO_2−δ_, 16.5 W m^−1^ K at RT) and conventional (Bi_0.52_Sb_1.48_Te_3_, 1.35 W m^−1^ K^−1^ at 310 K) TEs, which operate around RT, polymers show much lower κ values overall (e.g., PEDOT:PSS/EMIM-DCA, 0.30 W m^−1^ K^−1^ at 300 K and PEDOT:PSS, 0.52 W m^−1^ K^−1^ at 300K) [24,82]. This innate low κ of polymer TEs contributes significantly to the reasonable values reported for their *ZT* (0.1–0.75, Table 4), which are comparable to some typical conventional TEs (Table 2). Indeed, both acid-base treated PEDOT:PSS/EMIM-DCA (0.75) and PEDOT:PSS (0.42) have a greater *ZT* than both Pb_0.995_Sb_0.005_Te (~0.32) and Bi_2_Te_3_ (~0.25) at ~RT (Table 2). The overall performance of polymers at RT is similar to that of typical inorganic conventional TEs. However, many polymers have a much narrower temperature range and are usually restricted to applications from RT to a maximum of ~600 K.

### 3.2. Polymer TE Materials Used in Inorganic/Polymer Composites

**Figure 4:**
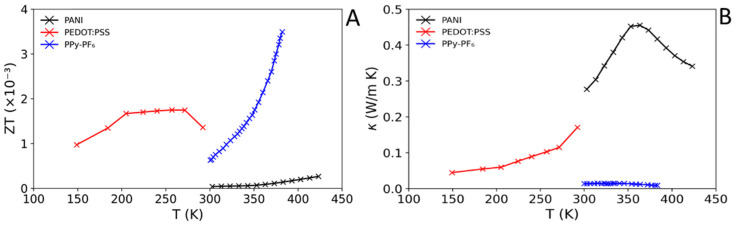
Comparison of *ZT* (**A**), and κ (**B**) for typical polymer TE materials used within inorganic/polymer composites as a function of temperature (K). PANl [129], PEDOT:PSS [130], and PPy-PF_6_ [132].

Conductive polymers, such as poly(3,4-ethylenedioxythiophene) (PEDOT), polyacetylene (PA), polyaniline (PANI), polypyrrole (PPy) and polythiophene (PTh) derivatives, are p-type thermoelectrics when oxidised [115]. Due to the flexible and elastic nature of polymers, thermoelectric developments, often in conjunction with doping and formulation including metal/organic fillers, have been driven by the creation of flexible, wearable electronic devices powered by body-heat alone [127,128]. In this Section, we discuss those polymers used within inorganic/polymer composites, and their TE properties. Table 5 summarises the TE parameters at *ZT*_max_ or at the maximum of PF, for those polymers used in inorganic/polymer composites. Figure 4 shows the *ZT* and κ of various polymers used within composites over their reported temperature ranges. Unlike leading polymer TEs in Table 4, polymers presented in Table 5 and Figure 4 are typical formulations that have not yet been fully optimised. 

**Table 5:** Thermoelectric properties of more typical polymer TEs used within inorganic/polymer composites at the stated temperature (*T*). P_3_HT = Poly(3-Hexylthiophene). *σ* is electrical conductivity, *S* is the Seebeck coefficient, PF is the power factor and κ is the thermal conductivity. All values are reported at the maximum of the figure of merit, *ZT*_max_. ^†^ Calculated from literature values.

Polymer	*T* (K)	σ	*S* (µV K^−1^)	PF (µW m^−1^ K^−2^)	κ	*ZT*_max_	Polymer Class
PANI [129]	423	~1.39	~40	~0.22 ^†^	~0.34	2.67 × 10^−4^	N
PEDOT:PSS [130]	270	~52	~12	~0.75 ^†^	~0.12	1.75 × 10^−3^	PTh
PEDOT:PSS [131]	298	1786	28.1	141	-	-	PTh
PPy-PF_6_ [132]	380	2.4	18.5	0.08	0.009	3.38 × 10^−3^	NAr
PPy-Tos [132]	380	20.7	14.9	0.46	0.18	0.97 × 10^−3^	NAr
PPy [132]	380	11.6	16.5	0.32	0.12	1.01 × 10^−3^	
PPy-HCl [132]	380	10.6	11.4	0.14	0.02	2.66 × 10^−3^	NAr
P3HT/Li + TBP [133]	333	~0.66	169	1.87	-	-	PTh

The two classes of polymers have different reported operating temperature windows: 300–450 K for N and NAr, and lower temperatures 150–300 K for the PTh polymer, PEDOT:PSS (Figure 4). 

When comparing σ for the more typical conductive polymers (σ = 0.60–1786 S cm^−1^, Table 5 and Figure 4), it is apparent that they have a generally much lower σ compared to typical inorganic materials (σ = 35–1000 S cm^−1^, Table 2). This is with the exception of PEDOT:PSS with a σ = 1786 S cm^−1^ comparable to the highest reported oxide TE, Na_x_CoO_2−δ_ (~1923 S cm^−1^ at 800 K, Table 1) and conventional TE, Bi_0.3_Sb_1.7_Te_3_ (~859 S cm^−1^ at 378K, Table 1). 

For polymers, thermopower values (*S*) are reported in the range 11–169 µV K^−1^ (Table 5, Figure 4), which is low in comparison to the range of typical conventional TEs (141–280 µV K^−1^, Table 2, Figure 3), but has some overlap with the range of typical oxide TEs (103–340 µV K^−1^, Table 2, Figure 3). The highest reported *S* is 169 µV K^−1^ at 333 K for P3HT/Li + TBP, which is larger than that of the n-type conventional TEs, Bi_2_Te_3_ (~−141 µV K^−1^ at 325 K) and Pb_0.995_Sb_0.005_Te (~−157 µV K^−1^ at 300 K) (Table 2, Figure 3). 

The power factors of polymers are situated in the range 0.08–141 µW m^−1^ K^−2^ (Table 5) with values between 0.08 and 0.50 µW m^−1^ K^−2^ being more typical. It is apparent that the typical PFs for polymers are far smaller than those reported for inorganic materials, Table 1 and Table 2, Figure 3. The most impressive PF reported is for PEDOT:PSS (141 µW m^−1^ K^−2^); however, this is still far lower than the ranges of PF values for both oxide (359–7690 µW m^−1^ K^−2^) and conventional TEs (578–5269 µW m^−1^ K^−2^). 

The thermal conductivity (Table 5, Figure 4B) of polymers is reported to be in the range 0.009–0.34 W m^−1^ K^−1^. These values are inherently low when compared to the κ ranges of both oxide (0.60–7.94 W m^−1^ K^−1^) and conventional (0.22–3.10 W m^−1^ K^−1^) TEs, which tend to be much higher (Table 1 and Table 2, Figure 3). At room temperature, higher values are usually observed for inorganic materials, particularly in oxide TEs as discussed in Section 2.2. The lowest κ = 0.009 W m^−1^ K^−1^ is shown by PPy-PF_6_ at 380 K. When inspecting the effect of temperature on κ (Figure 4B), the polymer classes, PTh and N/NAr, behave differently. For the PTh polymer, PEDOT:PSS, there is a steady increase in κ at T < 200 K followed by a sharper increase. In Figure 4B, for the N and NAr polymers, PANI and PPy-PF_6_, a similar effect is observed, where there is an increase in κ in the range 300–350 K. However, for both polymers there is a sudden sharp decrease in κ at ~360 K. 

The *ZT* values for polymers reported in Table 5 and Figure 4A, are all rather unimpressive sitting within the range 2.67 × 10^−4^–3.38 × 10^−3^. PPy derivatives report the highest *ZT* values, with PPy-PF_6_ displaying the highest *ZT* = 3.38 × 10^−3^ at 380 K. This is closely followed by the PTh-based polymer, PEDOT:PSS. The overall performance of *ZT* does not come close to matching conventional TEs at RT or indeed matching the performance of oxide TEs in general (Section 2.2). For *ZT* as a function of temperature, Figure 4A, there is a distinction between PTh and N polymers. The *ZT* values of PEDOT:PSS increase steadily from 150 to 270 K followed by a sharp decrease, while the N/NAr classes are reported at a higher temperature range (300–430 K) and show an overall increase in *ZT* with increasing temperature. 

### 3.3. Polymer/Carbon TE Composites 

**Figure 5:**
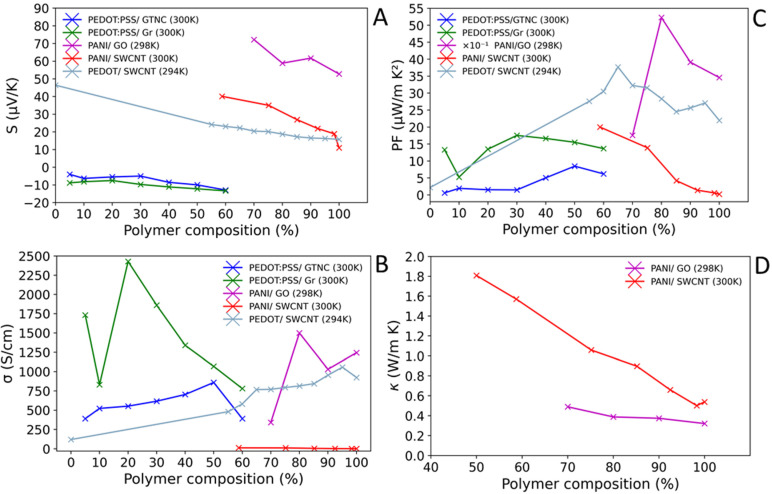
Comparison of TE parameters of polymer/carbon composites as a function of the polymer composition (%). (**A**) *S*. (**B**) σ. (**C**) PF. (**D**) κ. PANI/GO [141], PEDOT:PSS/GTNC [146], PEDOT:PSS/Gr [146], PEDOT/SWCNT [143], and PANI/SWCNT [157]. Note, values for (**C**) PANI/GO have been presented ×10^−1^.

The manufacturing of polymer/carbon composites TEs is much more commonly explored than that of inorganic/polymer composites. As we leave their detailed discussion to a number of reviews [37,127,128,134,135,136,137,138], while here we provide a general overview as the addition of carbon is an attractive avenue to improve performance of many composites, including inorganic/polymer composites. The combination of polymer materials with “fillers”, particularly highly conductive carbon and its allotropes, yields improvements in σ and *S* resulting in higher PFs. A number of reviews have been published on polymer/carbon composites [127,128,134]; however, here we will discuss the effect of combining carbon species with polymers in terms of TE properties for those compositions that are relevant for mixing with conventional or oxide TEs. In Table 6, polymer/carbon composites are presented in terms of their TE parameters at a stated temperature, while Figure 5 shows *σ*, *S*, κ and PF of polymer/carbon TE composites over their reported operating temperature ranges. 

**Table 6:** Thermoelectric properties of various polymer/carbon composites at a stated temperature (*T*). PVAc = Polyvinyl acetate, CQDs = carbon quantum dots, GTNC = graphene titanium nanocomposite, SWCNT = singled walled carbon nanotubes, DWCNT = double walled carbon nanotubes, rGO = reduced graphene oxide, GO = graphene oxide, Gr = graphene, TCPP = Meso-Tetra(4-carboxyphenyl)porphine. *σ* is electrical conductivity, *S* is the Seebeck coefficient, PF is the power factor, κ is the thermal conductivity and *ZT* is the figure of merit.

Composite	*T* (K)	σ	*S* (µV K^−1^)	PF (µW m^−1^ K^−2^)	κ	*ZT*	Polymer Class
p-type polymer/carbon composites							
PVAc/CQDs-C60 (15:5 ratio) [139]	300	~500	~65	210	~0.46	0.16	-
PANI (80 wt. %)/4D-Gr [140]	298	~394	~46	~82	-	-	N
PANI (80 wt. %)/GO [141]	298	~1500	~59	~521	~0.39	~0.40	N
P3HT (92 wt. %)/SWCNT [142]	298	~755	~51	~46	~0.13	-	PTh
PEDOT-Tos-PPP (65 wt. %)/SWCNT [143]	298	650	24.1	37.8	~0.4	-	PTh
PANI (58 wt. %)/SWCNT [144]	298	125	40	20	~1.44	-	N
PVAc (98 wt. %)/CQD [145]	298	30.3	71	15.2	~0.88	5 × 10^−3^	-
PEDOT:PSS (50 wt. %)/GTNC [146]	300	~890	~10	~8	-	-	PTh
PANI (50 wt. %)/rGO [147]	298	~18	32.64	2	~0.13	4.6 × 10^−3^	N
PEDOT:PSS (85 wt. %/GQDs [148]	298	71.2	14.6	~1.7		-	PTh
PVAc (60 wt. %)/SWCNT [149]	300	~900	~40	~1.44	~0.25	-	-
PVAc (5 wt. %)/Gr [150]	300	~29	~21	~1.24	-	-	-
P3HT (70 wt. %)/Gr [151]	298	~2	~36	~0.8	-	-	PTh
PEDOT:PSS (85 wt. %)/rGO [148]	298	~64	~11	~0.74	-	-	PTh
PEDOT:PSS (85 wt. %)/GO [148]	298	~59	~7	~0.3	-	-	PTh
PEDOT:PSS (97 wt. %)/rGO [152]	300	~637	~27	~46	-	-	PTh
PEDOT:PSS (40 wt. %)/DWCNT/TCPP [153]	298	~960	70	500	0.12	-	PTh
PTh (98.5 wt. %)/Gr [154]	298	1.8 × 10^−3^	-	-	~0.79	-	PTh
PANI/Gr/PANI/DWNT [155]	298	1080	130	1825	-	-	N
PANI/Gr-PEDOT:PSS/PANI/DWNT/PEDOT:PSS [156]	298	1900	120	2710	-	-	-
n-type polymer/carbon composites							
PVAc (20 wt. %)/GTNC [146]	300	~260	−42	47	2.9	4.8 × 10^−3^	-

The overall σ values (Table 6, Figure 5) of polymer/carbon composites are in a quite broad range, i.e., σ = 1.8 × 10^−3^–1900 S cm^−1^, with most common values in the 10^2^ S cm^−1^ range. This is a substantial improvement when compared to polymers alone (Table 4 and Table 5, Figure 4), and is indicative of the innate high σ of the carbon constituents. 

Many σ values reported are comparable to the higher-performing inorganic TEs (Table 1 and Table 2, Figure 3), e.g., Zn_0.97_Al_0.02_Ga_0.02_O (~570 cm^−1^) and nanostructured Bi_2_Te_3_ (~610 S cm^−1^). The σ of polymer/carbon composites are also very competitive with typical inorganic/carbon composites (Table 3), with some reported values exceeding the highest reported σ, Gr (0.6 wt. %)/La_0.067_Sr_0.933_TiO_3_ (1780 S cm^−1^). Meanwhile, a significant number of polymer/carbon composites have a very impressive σ > 10^3^ S cm^−1^ with some of them with a far greater σ than the highest reported conventional TE, Ba_0.08_La_0.05_Yb_0.08_Co_4_Sb_12_ (~1344 S cm^−1^), and similar to/exceeding the highest reported σ for oxide TE, Na_x_CoO_2−δ_ (~1923 S cm^−1^). For example, the PANI/Gr-PEDOT:PSS/PANI/DWNT/PEDOT:PSS (1900 S cm^−1^) and PANI (80 wt. %)/GO (1500 S cm^−1^) exhibit a far greater σ than that of Ba_0.08_La_0.05_Yb_0.08_Co_4_Sb_12_, with the former composite having a similar σ in value to Na_x_CoO_2−δ_. For σ as a function of polymer loading, Figure 5B, there is not a clear overall trend. This may imply that σ is highly dependent on the nature of the fillers and/or polymer, and the nanostructure/microstructure produced at the various loadings. At the extremes of material loadings, there is a tendency for less than optimal σ, implying that optimal loading will be a more balanced percentage composition of each constituent. For example, PANI/GO shows $\sigma_{max}$ = ~1499 S cm^−1^ at 80% polymer loading, while PEDOT:PSS/Gr shows maximum, $\sigma_{max}$ = ~2430 S cm^−1^ at 20% polymer loading.

The sign of *S* is positive for all but one composite (PVAc (20 wt. %)/GTNC), suggesting that p-type conductivity dominates polymer/carbon composites. Overall, the reported *S* values are rather low with typical values around *S* = 10^1^ µV K^−1^. The reported range of *S* is 6–130 µV K^−1^ with the highest thermopower belonging to the PANI/Gr/PANI/DWNT composite (130 µV K^−1^), which is competitive with some conventional TEs at RT, e.g., Pb_0.995_Sb_0.005_Te (−154.24 µV K^−1^). When comparing *S* of polymer/carbon composites to typical polymers (Table 5), there is an overall slight improvement in *S*, with *S* values more comparable to the highest performing polymers (Table 4). The overall verdict on *S* for polymer/carbon composites is that currently they typically do not compare to inorganic TEs. 

Within Figure 5A, there is an overall decrease in *S* as the percentage of polymer increases. This may be explained by the increase in charge carrier mobility (μ) with increasing polymeric percentage. This is further supported by the increase in σ in line with wt. % of polymer observed for some polymer/carbon composites (Figure 5B). 

The power factor of polymer/carbon composites displays a wide range of values, 0.3–2710 µW m^−1^ K^−2^, but with the majority lying within the 1–100 µW m^−1^ K^−2^ range. There is a general improvement in PF compared to the typical polymer constituents (Table 5), with values for the polymer/carbon composites more comparable to the state-of-the-art polymers (Table 4). Furthermore, most composites do not compare to inorganic TEs (Table 1 and Table 2, Figure 3) and their composites of carbon (Table 3) in terms of PF. However, there are some impressive PFs reported, e.g., PANI/Gr-PEDOT:PSS/PANI/DWNT/PEDOT:PSS (2710 µW m^−1^ K^−2^), PANI/Gr/PANI/DWNT (1825 µW m^−1^ K^−2^), PANI (80 wt. %)/GO (~521 µW m^−1^ K^−2^), as these compare to several inorganic TEs (Table 1 and Table 2, Figure 3). The dependence of PF on polymer loading, Figure 5C, does not appear to have a clear trend. Nevertheless, the PF *vs.* polymer loading curves display maxima between the 50–80% polymer loading range. For PEDOT:PSS, the optimal range appears to be within 50–60% polymer loading, while PANI/GO shows its PF maximum at ~70% polymer loading. As PF is a combination of both *S* and σ, improvements can be achieved by balancing both parameters in terms of charge carrier mobility (μ, Equations (4)–(6)) by optimising the polymer loading.

It is important to note that polymers such as PVAc, which by itself is not a thermoelectric material and has a low σ at RT, [158,159], can also be combined with carbon and its allotropes to display TE properties [149]. An example of this is the PVAc/CQDs-C60 composite (15:5 ratio) reported in Table 6, with a reasonable PF = 210 µW m^−1^ k^−2^ along with *ZT* = 0.16, in part due to its low κ = 0.46 W K^−1^ m^−1^. A justification for using non-TE polymers in polymer/carbon composites is that they are cheap, widely available, flexible and easy to work with [160].

On addition of carbon fillers, similar or slightly larger κ values are noted when compared to the polymer constituents alone (Table 4 and Table 5). However, the increase in κ due to filler content is often negligible due to the increase in both σ and *S*. κ is highly dependent on the polymer loading, Figure 5D. Decreasing filler loading, i.e., high polymer loading, shows a tendency to reduce thermal conductivity of the composite material, which may be explained by the more thermally conductive nature of carbon allotropes. The increase in *S* and σ leads to some rather impressive *ZT* values at RT for the composite materials, sometimes of many orders of magnitude when compared to their constituent materials. For example, PANI has *ZT*_max_ = 2.7 × 10^−4^ at 420 K (Table 5) while Graphene-oxide/PANI (80 wt. %) results in *ZT*_max_ = 0.40 at RT [129,141]. This significantly larger improvement in *ZT* is mostly attributed to the much improved σ. Although few *ZT* values have been reported for polymer-carbon composites, a few do compete with a number of typical inorganic TEs (Table 1 and Table 2, Figure 3), namely, PVAc/CQDs-C60 (15:5 ratio) (*ZT* = 0.16) and Graphene-oxide/PANI (80 wt. %) composites. 

The TE parameters of polymer/carbon composites do not compare to those of inorganic TEs (Table 1, Table 2 and Table 3, Figure 3). For an effective viability of TE composites, *ZT* must be >1. However, lower power applications may still be applicable such as “wearable” technologies [127,128]. Polymer/carbon composites are more suitable for lower temperature range applications, i.e., 298.15–434.15 K, due to their polymer constituents usually possessing a low decomposition temperature (Section 5). However, these polymer/carbon composites should be considered when engineering inorganic/polymer composites due to the benefits that the carbon filler has on *S*, κ and σ. Generally, the combination of polymer/carbon composites with oxide or conventional inorganic materials should aim to maintain a balance between improvements on both *S* and σ while retaining the low κ innate to polymers. 

## 4. Inorganic/Polymer Composites

The nanoscale structure is fundamental to TE performance. Research focusing on structural features such as defect clusters, voids, and interfaces often yields promising results in oxide and conventional TEs [161,162]. The formation of interfaces in composites is currently exhibiting great potential within many fields from catalysis to medicine. Interfaces interrupt the phonon mean free path and/or may create quantum confinement effects, which lead to the enhancement of TE properties [134,148,163]. The formation of interfaces in composites could be broadly placed in two phonon engineering strategies, namely “increased scattering” and “altered dispersion relations”. Inorganic/polymer composites see application in flexible/wearable devices, as discussed in Du et al. [164]. However, the review of Du et al. focusses on the complexity and performance of the TE flexible/wearable devices which mostly consist of telluride/polymer composites and their addition to textiles. In this review, we target inorganic/polymer composites with focus on the performance of the constituent materials, the key-performing conventional/polymer composites and their recent advances.

One of the challenges of creating composite materials is maintaining or improving both *S* and *σ*. As the value of *S* is mainly determined by the energy of the Fermi level and the position of the band edge [165], the formation of nanostructures and the creation of composites can lead to large electronic changes. For example, the introducing extrinsic inclusions/additions and grain-boundaries in inorganic materials may alter the Fermi level relative to the conduction band. This could increase the bandgap and decrease conductive channels, thus leading to a reduction in *S* and *σ*. The decrease in *σ* can further be explained as a reduction in μ (Equation (6)) [166,167]. An ideal system would have a Fermi level located in proximity to the bottom of the conduction band as this would lead to a larger *σ*. In theory, the band structure of a composite may be improved by the introduction of a higher abundance of highly conductive fillers such as graphene, polymers etc.

Here, we present inorganic/polymer composites which are relevant to TE applications. Within Section 4.1, representative examples of conventional/polymer composites, including some containing carbon, are briefly discussed and compared to their conventional and polymer individual constituents. The discussion then continues towards the main focus of this review (Section 4.2), i.e., the oxide/polymer composites, including some carbon composites for comparison. The discussion includes the assessment of the composites’ TE properties and their constituent materials, and suggestions for their improvement. For a more detailed discussion on carbon composites see Section 2.3 and Section 3.3. 

### 4.1. Conventional/Polymer Composites 

The combination of conventional TE materials and polymers will be briefly discussed along with a comparison made to both the individual polymer and the conventional TEs. For an in-depth discussion of the topic, we refer the reader to existing reviews [127,134,168,169]. Table 7 summarises the TE properties of conventional/polymer composites at a stated temperature. 

Nanostructuring (grain boundary engineering, low dimensionality, etc.) of conventional TE materials have yielded great improvements, often showing an increase in σ and a reduction in κ. This is particularly apparent when comparing nanostructured Bi_2_Te_3_ reported in Table 2 (κ = 0.59 W K^−1^ m^−1^ and *σ* = ~608 S cm^−1^ at 400 K) to its bulk form (1.29 W K^−1^ m^−1^, ~483 S cm^−1^ and *σ* = ~483 S cm^−1^ at 325 K, Table 2) [75,76]. Indeed, the addition of polymers to conventional TEs aims to have a similar effect to nanostructuring. 

When comparing several composites (Table 7) to their conventional TE constituents (Table 1 and Table 2), there is an overall increase in σ, which can be attributed to the conductive nature of the polymer species used within the composite. σ for the reported conventional/polymer composites sit within a broad range (1.9–945 S cm^−1^) with many around 10^2^ S cm^−1^. When comparing PEDOT:PSS (5% DMSO)/Bi_2_Te_3_ composite (945 S cm^−1^ at 298 K) to the conventional Bi_2_Te_3_ (~483 S cm^−1^ at 325 K, Table 2), the addition of the polymer yields an increase in conductivity of 462 S cm^−1^. Indeed, when PEDOT is also combined with CNT, creating the composite PEDOT/CNT/Bi_2_Te_3_ (Table 7), an increase of ~433 S cm^−1^ is also shown compared to conventional Bi_2_Te_3_. It is apparent that when comparing between composites containing either class of polymer, N and PTh, the N class suffer from much lower *σ* (σ_max_ = 102 S cm^−1^, PANI (30 wt. %)/Te nanorods) compared to the PTh class (σ_max_ = 945 S cm^−1^, PEDOT:PSS (5% DMSO)/Bi_2_Te_3_). The few composites reported with high polymer loadings (>60% polymer) show a lower σ compared to their conventional constituents. When comparing σ of PANI (70 wt. %)/Bi_2_Te_3_ composite (11.5 S cm^−1^) to pristine PANI (139.0 S cm^−1^, Table 4) and conventional Bi_2_Te_3_ (~483 S cm^−1^), there is a clear disparity, with the composite possessing a much lower σ. For conventional/polymer composites, the nature of σ seems dependent upon the nature of the composite’s constituents, with higher polymer loadings linked to lower σ, due to more significant alterations to the band structure, or the interruption of conductive channels compared to the pristine conventional material [167]. Conversely, σ of conventional materials may be improved upon with small additions of polymer, due to the retainment of the band structure of the conventional material and/or overlap/alteration of the materials bandgap and Fermi region, as demonstrated in a recent theoretical study involving compositing a semi-conductive inorganic material with Graphene [178,179].

When considering the majority of conventional/polymer composites across both polymer classes (Table 7), it is apparent that a number of the values of the Seebeck coefficient are significantly lower than those reported for conventional materials (Table 2, *S* = 210–1000 µV K^−1^), and are indeed more comparable to most polymers (Table 5, ~10^1^ µV K^−1^). All of the PTh-based composites have high σ but low *S* values. Of the N-based composites, those containing PANI have a more balanced σ and *S*. Again, the relationship between *S* and σ is attributed to the nature of the polymer, the polymer–inorganic interface and the intricate balance between *S* and σ. For a more detailed discussion, please refer to Section 3.1 and Section 1. The reported range of *S* for the conventional/polymer composites is 20–190 µV K^−1^, with all composites having a p-type behavior. This differs from many of the conventional TEs used within the conventional/polymer composites, which are usually n-type when in their bulk form (Table 1 and Table 2). This is perhaps due to the ratio of polymer to conventional materials, as all the tabulated (Table 7) composites, which report composition and contain n-type conventional materials, have high ratio of polymer to conventional material, e.g., PANI (70 wt. %)/Bi_2_Te_3_. The highest performing conventional/polymer composites in terms of *S* are PANI (70 wt. %)/Bi_2_Se_3_ (~188 µV K^−1^) followed by PEDOT/CNT/Bi_2_Te_3_ (~122 µV K^−1^). The latter has an *S* lower in magnitude than that reported for its conventional constituent Bi_2_Te_3_ (Table 2, ~141 µV K^−1^). Conversely, the addition of conventional TEs to polymers can considerably increase the magnitude of *S*, for example, PANI (70 wt. %)/Bi_2_Se_3_ composite has indeed a higher *S* than PANI (39.5 µV K^−1^), Table 5. 

There are some striking PF values reported in Table 7, such as PEDOT/CNT/B_i2_Te_3_ (1400 µW m^−1^ K^−2^), which is far greater than any of those reported for the state-of-the-art polymers (Table 4, PF_max_ = 1270 µW m^−1^ K^−2^ for PP-PEDOT:Tos), and a number of the state-of-the-art inorganic materials (Table 1). Although the range of PF for the reported conventional/polymer composites varies substantially (PF = 1.55–1400 µW m^−1^ K^−2^), the highest performing composites (where PF = ≥10^2^µW m^−1^ K^−2^) are observed over almost all of the polymer loadings (5–80 wt. %) and seem to be highly dependent upon the conventional materials present. When comparing between polymer classes, PTh class report much higher PF (PF_max_ = ~1393 µW m^−1^ K^−2^, PEDOT/CNT/Bi_2_Te_3_) than the N class (PF_max_ = 107.67 µW m^−1^ K^−2^, PANI (70 wt. %)/Bi_2_Se_3_), although the PTh front-runner composite does also contain carbon. Overall, the PF values of the conventional/polymer composites are more comparable to the state-of-the-art polymers (Table 4) than conventional TEs (Table 1 and Table 2). The addition of conventional TEs to conventional/polymer composites results in an improvement of PF values compared to those reported for typical polymers (Table 5, Figure 4), bringing their PF values in line with those of the state-of-the-art polymers (Table 4). Indeed, several conventional/polymer composites express a greater PF than those reported for inorganic/carbon composites (Table 3). When observing Figure 5C there seems to be an overall increase in PF in line with temperature, for the polymer/carbon composites shown. For the examples shown within Figure 5C, there seems to be an overall increase in PF as a function of temperature. 

The thermal conductivity (κ) is inherently low for all conventional/polymer composites, with those in Table 7 reporting κ = 0.2–0.96 W m^−1^ K^−1^ with no clear differences between composites containing either N or PTh classes of polymers. The majority of κ values for the composites are lower than the typical values for the state-of-the-art inorganic TEs (Table 1). There is a significant reduction in κ when compared to conventional TEs, e.g., when comparing PANI (70 wt. %)/Bi_2_Te_3_ (0.11 W m^−1^ K^−1^) to its inorganic constituent Bi_2_Te_3_ (1.29 W m^−1^ K^−1^, Table 2). The impressive κ for PANI (70 wt. %)/Bi_2_Te_3_ is indicative of the high polymer loading, and even when compared to PANI alone (0.34 W m^−1^ K^−1^, Table 5) a significant reduction in κ is also shown, perhaps attributed to the creation of material interfaces brought about by compositing. It is also the case that a number of PEDOT containing polymer composites show an increase in κ, for example when comparing PEDOT:PSS (80 wt. %)/SnSe nanosheet (0.36 W m^−1^ K^−1^) to PEDOT:PSS (0.12 W m^−1^ K^−1^, Table 5). In general, the resultant κ of nanocomposites is dependent upon the nature of their constituents, their respective loadings and the characteristics of the resultant nanostructure. 

The *ZT* reported for conventional/polymer composites vary significantly, i.e., 4.3 × 10^−3^–0.49, with the majority of reported *ZT* values (Table 7) being much lower than those reported for conventional TEs. *ZT* for composites are more in line with those reported for the majority of the state-of-the-art polymers (Table 4), with the N class (PANI) only expressing slightly lower *ZT* values (~4.3 × 10^−3^–0.18), compared to the PTh class (0.2–0.49). The highest *ZT* belongs to PEDOT/CNT/Bi_2_Te_3_ (0.49 at 325 K), which is a rather impressive increase compared to typical Bi_2_Te_3_ (0.25 at 325 K, Table 2). This increase can be attributed to a greatly improved electrical conductivity while maintaining a similar *S* compared to Bi_2_Te_3_. PANI (70 wt. %)/Bi_2_Te_3_ expresses a low *ZT* of 4.3 × 10^−3^ at 300 K, far less than that of Bi_2_Te_3_ (Table 2) but still slightly higher than PANI alone (2.67 × 10^−4^ at 420 K, Table 5). In general, all the *ZT* values reported are greater than typical polymers (Table 5).

### 4.2. Oxide/Polymer Composites 

The addition of polymers such as PANI, PEDOT:PSS, etc., to oxide materials is expected to yield similar improvements to the more explored science of creating composites via the addition of carbon allotropes to inorganic materials. In Section 2.3, the creation of inorganic/carbon composites has been shown to broaden the operating temperature range, and a reduced temperature at which maximum *ZT* is achieved, allowing for possible low-temperature applications (RT) [31]. Although current research on polymer-based composites is limited, there have been a few compositions exhibiting great promise. This section aims to discuss the work conducted so far. 

**Figure 6:**
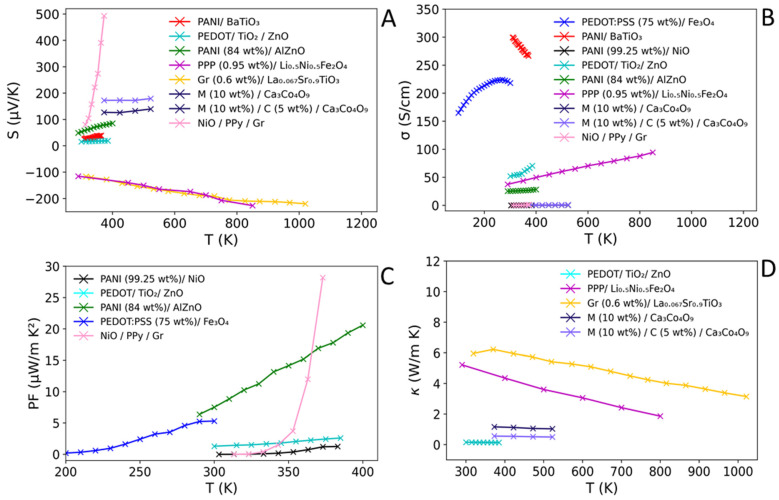
Comparison of TE parameters of oxide/polymer composites as a function of temperature (T). (**A**) *S*. (**B**) σ. (**C**) PF. (**D**) κ. PANI/BaTiO_3_ [188], PEDOT/TiO_2_/ZnO [189], PANI (84 wt. %)/AlZnO [190], PPP (0.95 wt. %)/Li_0.5_Ni_0.9_Fe_2_O_4_ [196], Graphene (0.6 wt. %)/La_0.067_Sr_0.9_TiO_3_ [31], PEDOT:PSS (75 wt. %)/Fe_3_O_4_ [192], PANI (99.25 wt. %)/NiO [191], M (10 wt. %)/Ca_3_Co_4_O_9_, M (10 wt. %)/C (5 wt. %)/Ca_3_Co_4_O_9_ [194], and NiO/PPy/Gr [195]. M = Matrimid, C = carbon black.

From the available literature, it is clear that the creation of oxide/polymer composites is relatively underrepresented within TE research, with only a handful of review articles briefly mentioning them [164,180,181]. Oxides also show promising results for other energy applications such as supercapacitors, sensors and photovoltaics [182,183,184,185,186,187]. Table 8 summarises the TE parameters of oxide/polymer TE composites at a stated temperature, whilst Figure 6 shows *σ*, *S*, κ and PFs of various oxide/polymer TE composites over their reported temperature ranges. Here, a discussion takes place around the rationale of compositing: TE parameters of composites are compared to their individual constituents (oxide and polymer TEs), and the addition of carbon fillers is briefly discussed. 

One could infer the optimal temperature operating range of the oxide/polymer composites using Figure 6. The temperature range is highly indicative of the nature of the constituent polymer within the oxide/polymer composite, as discussed in Section 3. The majority of oxide/polymer composites operate within a small thermal window, RT − 400 K, while those containing either PPP or graphene (Gr) alone operate from RT to high temperatures, >800 K. The oxide/polymer composite containing PPP and Gr, nevertheless, still suffer from degradation of TE properties at >700 K in air [198]. On the whole, the oxide/polymer composite temperature ranges reported are limited to low-to-mid temperatures (300–600 K), implying that the ideal operating temperature lies within this and hence would be more suited for ambient and low-grade heat recovery and conversion. For example, body-heat generators or perhaps recovery of heat generated by electronics or low-grade industrial sources. It could be suggested that the optimisation of polymer-loading could be used to fine-tune such operating temperature ranges, with larger loadings becoming more optimal for lower temperatures.

Electrical conductivity, σ, is an important factor within thermoelectric materials with research often aiming to increase it, while retaining a low κ and a large *S*. If we compare the σ values of oxide/polymer composites to those of polymers (Table 4 and Table 5, Figure 4), on average the σ values remain similar or are lower, depending on the composition of the oxide/polymer composites. This is with the exception of several PANI-based composites, e.g., PANI/BaTiO_3_, which shows an increase in $\sigma_{max}$ of ~268 S cm^−1^ when compared to PANI alone (1.39 S cm^−1^, Table 5). When oxide/polymer composites are compared to their oxide constituents, there is a large variation in σ dependent on the composition of the composites. In general, Table 8 shows an overall decrease in σ for oxide/polymer composites compared to their oxide constituents (Table 2). As direct comparison between different classes of TE materials is difficult, due to their potentially different operating temperatures, we only compare the maximum performance. An example of the large variation within σ depending upon the oxide/polymer composition is evident within the PANI/BaTiO_3_ and PANI (84 wt. %)/Al_0.4_ZnO composites. PANI/BaTiO_3_ (270 S cm^−1^) has a greater σ than doped BaTiO_3_, σ = 63 S cm^−1^, whereas PANI (84 wt. %)/Al_0.4_ZnO (25 S cm^−1^) shows a smaller σ compared to Zn_0.998_Al_0.02_O, ~1370 S cm^−1^. 

Figure 6B shows that there is little temperature dependence on the magnitude of σ for most oxide/polymer composites, and therefore the thermal effects on σ are heavily dependent upon the composition of the material. For example, 0.95 wt. % PPP/Li_0.5_Ni_0.5_Fe_2_O_4_ shows an almost linear increase in σ with increasing temperature, while an inverse-parabola-like curve is shown for 0.75 wt. % PEDOT:PSS/Fe_2_O_4_ consisting of an initial increase in σ up to ~270 K after which a steady decrease is observed. When comparing the oxide/polymer composites based on the polymer class (i.e., NAr, N and PTh), the PTh class containing composites express the highest σ values (PEDOT:PSS (5 wt. %)/GINC), followed by the N class (PANI/BaTiO_3_), and the NAr class (NiO/PPy/Gr, Matrimid (10 wt. %)/C (5 wt. %)/Ca_3_Co_4_O_9_).

The addition of graphene and/or other highly conductive fillers to the oxide/polymer composites could be a promising strategy to improve σ. Another strategy may be to increase the loading of the inorganic material, and hence having a lower polymer percentage. However, this may also lead to an increase in thermal conductivity, which may be negated by the overall improvement of *S* and σ anyway, and hence may speculatively yield much greater potential as promising TE composites. Overall, σ is highly dependent upon the composition of the composite, and of course temperature. 

The majority of the oxide/polymer composites discussed retain a p-type conductivity native to the polymers. The few showing n-type conductivity contain either low weights of the polymer or incorporate nanoparticles, e.g., (PPP 0.95 wt. %)/Li_0.5_Ni_0.5_Fe_2_O_4_ and PANI (80 wt. %)/SrTiO_3_). On the other hand, the p-type oxide/polymer composite, PANI (84 wt. %)/Al_0.4_ZnO (53.6 µV K^−1^), contains an n-type oxide, Zn_0.998_Al_0.02_O (*S* = ~−150 µV K^−1^, Table 2) [190]. This implies that varying the polymer loading could lead to changes in the conduction regimes. It is expected that the addition of lower concentrations of polymers to inorganic oxides would increase *S* towards the p-regime, while the addition of oxides to high polymer loadings would decrease or increase the conductivity regime present within the polymer depending on the nature of the oxide material. This influence of different loadings is observed for the oxide/polymer composite, PANI (84 wt. %)/Al_0.4_ZnO, as when compared to PANI, ~40 µV K^−1^ at 420 K (Table 5, Figure 6), there is an increase in *S*. However, the literature reports *S* = 9 µV K^−1^ for PANI at 300 K [129], thus suggesting that the increase in *S* of the composite arises from the mixture of the two constituents and not on the temperature of the measurement. 

The n-type composite material containing the NAr polymer PPP, 0.95 wt. % PPP/Li_0.5_Ni_0.5_Fe_2_O_4_, shows the highest thermopower (*S*), comparable to the carbon-based composite Graphene (0.6 wt. %)/La_0.067_Sr_0.9_TiO_3_ (Table 3, Figure 6), with a similar trend over the range 300–850 K. In terms of overall thermopower, polymer/oxide composites show higher performance when the polymer belongs to the NAr, N, and PTh classes, respectively. For p-type composites, PANI (N polymer) (84 wt. %)/Al_0.4_ZnO shows the highest *S* value followed by PANI/BaTiO_3_. The magnitude of *S* for those within the p-regime is significantly lower, never exceeding 100 µV K^−1^. For both conductivity regimes (n and p), there is an increase in the magnitude of *S* as temperature increases. To summarise, the thermopower of oxide/polymer composites is comparable to those reported for polymers (Table 4 and Table 5, Figure 6) and remain similar, or is greatly improved, depending on the composition. For example, PANI (99.25 wt. %)/NiO has a large *S* (331 µV K^−1^ at 383 K) while PANI alone is 39.5 µV K^−1^ at 420 K. When compared to oxide materials (Table 1 and Table 2, Figure 3), the magnitude of *S* is greatly reduced, for example comparing the oxide, Zn_0.998_Al_0.02_O ( −150 µV K^−1^) to PANI (84 wt. %)/Al_0.4_ZnO (53.6 µV K^−1^). 

PF increases with temperature with the exception of 0.75 wt. % PEDOT:PSS/Fe_2_O_4_, which levels out and starts to decrease above ~290 K (Figure 6C). The largest PF is observed for PANI (84 wt. %)/Al_0.4_ZnO, 20.60 µW m^−1^ K^−2^ at 300 K [190]. There is no clear trend in PF when considering the different polymer classes within the oxide/polymer composites. When the oxide/polymer composites are compared to their constituent polymers (Table 4 and Table 5), there appears to be no straightforward trend in PF values for oxide/polymer composites compared to their constituent polymers. The majority of the oxide/polymer composites retain similar or have lower PF compared to the polymer constituents, e.g., PEDOT:PSS (75 wt. %)/Fe_3_O_4_ (5.3 µW m^−1^ K^−2^) has a much smaller PF compared to PEDOT:PSS (141 µW m^−1^ K^−2^, Table 5) at RT. However, specific oxide/polymer composites have a higher PF when compared to their polymer constituent, e.g., NiO/PPy/Gr (~28 µW m^−1^ K^−2^ at 373 K) compared to PPy (0.32 µW m^−1^ K^−2^ at 380 K). In comparison to oxide materials (Table 1 and Table 2, Figure 3), polymer/carbon (Table 3) and many conventional/polymer composites (Table 7, Figure 6), the PF values of oxide/polymer composites are generally much lower. However, the oxide/polymer composite, Matrimid (10 wt. %)/C (5 wt. %)/Ca_3_Co_4_O_9_ (~62 µW m^−1^ K^−2^) in Table 8, shows great promise, followed by PEDOT:PSS (5 wt. %)/GINC (~52 μW m^−1^ K), as their PF values are in the range of those of conventional/polymer composites. The carbon filler-containing perovskite oxide composite, Graphene (0.6 wt. %)/La_0.067_Sr_0.933_TiO_3_ (Table 3, Figure 6), shows the greatest PF of any composite overall, which could be attributed to the presence of the highly conductive graphene. It is therefore suggested that a combination of both polymer and highly conductive carbon filler could be a promising route to improve PF. 

Thermal conductivity (κ) in Figure 6D is one of the most important limiting factors for oxide inorganic TE materials (Section 2). An ideal TE material would have a low κ over its whole temperature range, allowing for PF to be a more realistic expression of the overall power output. An issue with oxides and hence oxide/polymer composites is the variance of κ with temperature due to the dynamics of the lattice and subsequent phonon–phonon interactions. Lattices consisting of a layered structure, or those with many vacancies, tend to have a decreased κ due to phonon dispersion at the interface with the void. The introduction of extrinsic defects, particularly those that introduce rattling modes into the environment, act to reduce κ. Overall, an ideal lattice would adopt a “phonon-glass, electron-crystal” approach, which is described in detail within prior literature [199]. Although, there is a lack of data for some of the oxide/polymer composites in Table 8, the oxide/polymer composites with the smallest κ values, regardless of the composition, are those containing PEDOT, followed by those containing Matrimid. This differs from conventional/polymer composites (Table 7), where composites containing PANI exhibit the lowest κ. The lowest κ is reported for PEDOT/TiO_2_-ZnO, 0.13 W K^−1^ m^−1^ at 383 K, which is a significant reduction when compared to the inorganic ZnO, 7.6 W K^−1^ m^−1^. The overall trend shows that κ is reduced within polymer/oxide composite materials when compared to individual oxide TEs, while values remain similar, or slightly larger, than those reported for the individual polymers. 

For oxide/polymer composites which report *ZT* values, they are significantly low more comparable to those of polymers. These *ZT* values are similar or slightly improved compared to their polymer constituents (Table 5), e.g., PANI (*ZT* = 2.7 × 10^−4^ at 420 K) compared to PEDOT/TiO_2_-ZnO (ZT = 7.5 × 10^−3^ at 383 K).

The composites Matrimid (10 wt. %)/Ca_3_Co_4_O_9_ and Matrimid (10 wt. %)/C (5 wt. %)/Ca_3_Co_4_O_9_ allow us to compare between a composite with and without a carbon filler [194]. In Figure 6A when comparing the values of *S* at 523 K, where *S* = ~140 µV K^−1^ and ~178 µV K^−1^ for the composite containing no filler and filler respectively, a reasonable improvement of 38.6 µV K^−1^ is observed with the addition of carbon-black (5 wt. %). This is perhaps attributed to the greater conductivity of this constituent. This is supported by σ, Figure 6B, where also at 523 K, an improvement of ~0.68 S cm^−1^ is shown on the addition of carbon-black (5 wt. %) yielding an σ = ~1 S cm^−1^, a great improvement compared to the oxide/polymer composite without filler σ = 0.335 S cm^−1^. The κ is also shown to decrease, Figure 6D, where the κ = ~0.49 W K^−1^ m^−1^ at 523 K for the composite with filler, while κ = ~1.03 W K^−1^ m^−1^ at 523 K for that without. This results in a PF = 3.17 μW m^−1^ K^−2^ at 523 K for the filler containing material, a dramatic improvement upon the no filler composite, where PF = ~0.6 μW m^−1^ K^−2^. Hence providing evidence that the addition of filler to polymer composite materials can indeed improve TE properties. 

In general, strategies for improving oxide TEs aim to lower κ while retaining a reasonable *S* and σ. The introduction of a polymer alone tends to inhibit both σ and *S*. Therefore, the addition of both highly conductive filler and polymer to a high loading of oxide may be the recommended route to gain the most benefit from the creation of a composite. This should ensure a balance between all parameters and provide the most benefits from the disruption of phonon modes introduced by synergistic effects of the material’s interfaces.

## 5. Conclusions 

Oxide materials usually exhibit their optimal performance at high temperatures, whereas most conventional materials usually cannot operate within this range (>~700 K) [200]. If the optimum performance of oxides could be lowered, they could replace conventional materials and also fit within the ideal range of TE polymers. Overall, the addition of both carbon and polymer yields the most promise. The operating temperature range of oxide/polymer composites (with and without fillers) is still limited to lower temperatures like their polymer counterparts, with TE parameters varying considerably across their reported temperature ranges, suggestive of a very narrow window of maximum performance. 

**Figure 7:**
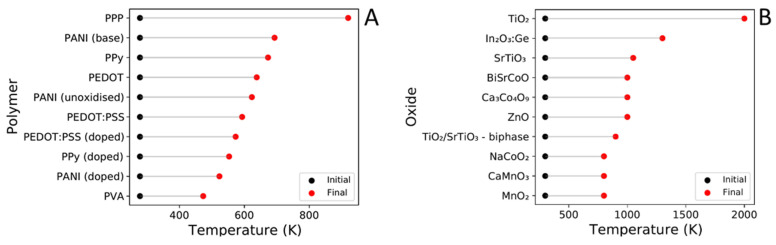
Reported temperature ranges of organic polymers (**A**) and oxides (**B**) in air as a function of temperature (K). (**A**) PPP [202], PANI (Base) [206], PPy [207], PEDOT [208], PANI (unoxidised) [209], PEDOT:PSS [208], PEDOT:PSS (doped) [208,210], PPy (doped) [207], PANI (doped) [206], and PVA [211]. (**B**) TiO_2_ [212], In_2_O_3_:Ge [80], SrTiO_3_ [213], BiSrCoO [80], Ca_3_Co_4_O_9_ [214], ZnO [23], TiO_2_/SrTiO_3_–biphase [215], NaCoO_2_ [24], CaMnO_3_ [216], and MnO_2_ [217].

Figure 7 is a graphical representation of the temperature ranges of polymers and oxides. Polymers (Figure 7A) have a lower and narrower operating temperature range, ~273–700 K in comparison to oxides, ~RT − ≥ 1000 K (Figure 7B), with the exception of PPP that shows a much larger range before thermal degradation (~273 − <1000) K [201].

When pairing materials for the generation of composites, the temperature range of each constituent may need careful consideration. The vast majority of polymers operate more effectively in the mid-to-lower temperature ranges reported by oxides (<600 K); however, there are some exceptions such as PANI (base), PEDOT and PPy that are stable over a larger portion of the oxide temperature range (<800 K). Therefore, the exclusive use of doped polymers for applications at ~RT − 320 K may be suitable, while the polymer PPP may be suited for higher temperature applications (>700 K) or in circumstances where there is a high variation in temperature range. 

The additions of carbon-based fillers to a polymer/oxide composite may also impact the temperature range of stability. It could also bring the optimal operating temperature range down to lower temperatures opening up the operating temperature window of the individual oxide [32,95]. Manipulation of the polymer structure via substitution or addition reactions, i.e., functionalisation of the polymer, is also known to alter the temperature of thermal degradation [202,203,204]. This effect is also observed when polymers are doped [205].

## Figures and Tables

**Table 4:** Thermoelectric properties of the highest performing polymers at a stated temperature (*T*). P_3_HT = Poly(3-Hexylthiophene), PEDOT = poly(3,4-ethylenedioxythiophene), PA = polyacetylene, PANI = Polyaniline, Ppy = Polypyrrole, PSS = Polystyrene sulfonate, Tos = Tosylate, EMIM-DCA = 1-Ethyl-3-methylimidazolium dicyanamide, FTS = Tridecafluoro-1,1,2,2-tetrahydrooctyl)trichlorosilane and PBTTT = Poly[2,5-bis(3-tetradecylthiophen-2-yl)thieno[3,2-*b*]thiophene]. PzDPP = pyrazine-flanked diketopyrrolopyrrol. σ is electrical conductivity, *S* is the Seebeck coefficient, PF is the power factor and κ is the thermal conductivity. All values are reported at the maximum of the figure of merit, *ZT*_max_. ^†^ Calculated from literature values.

Polymer	*T* (K)	σ	*S* (µV K^−1^)	PF (µW m^−1^ K^−2^)	κ	*ZT*_max_	Polymer Class
p-type polymers							
PEDOT:PSS/EMIM-DCA [116]	300	1600	65	754	0.30	0.75	PTh
PEDOT:PSS [109]	300	~880	~70	~460	0.52	~0.30	PTh
PP-PEDOT:Tos [117]	300	~930	~117	1270	-	-	PTh
P3HT [118]	365	320	~55	46 ^†^	0.23	~0.10	PTh
PANI [119,120]	300	220	~20	~11	-	6.1 × 10^−3^	N
PBTTT/FTS [121]	300	1000	33	110	-	-	PTh
n-type polymers							
FBDPPV [122]	300	~6	−198	25.5	-	-	-
ClBDPPV [122]	300	~4	−220	16.5	-	-	-
P(PzDPP-CT2) [123]	300	~4	~−378	57.3	-	-	-
UFBDPPV/TAM [124]	300	22.5	−198	80	-	-	-

**Table 7:** Thermoelectric properties of representative conventional/polymer composites at a stated temperature (*T*). PAA = poly(acrylic acid). *σ* is electrical conductivity, *S* is the Seebeck coefficient, PF is the power factor, κ is the thermal conductivity, and *ZT* is the figure of merit. ^†^ Calculated from literature values.

Composite	*T* (K)	σ	*S* (µV K^−1^)	PF (µW m^−1^ K^−2^)	κ (W m^−1^ K^−1^)	*ZT*	Polymer Class
PEDOT/CNT/Bi_2_Te_3_ [76]	325	~915	~123	~1393	~0.96	~0.49	PTh
PEDOT:PSS (80 wt. %)/SnSe nanosheet [170]	300	~320	~110	~400	~0.36	0.32	PTh
PEDOT:PSS/PAA/Bi_0.4_Te_3_Sb_1.695_ (95 wt. %) [171]	300	380	79	~240	0.36	0.20	PTh
PANI (70 wt. %)/Bi_2_Se_3_ [172]	410	30.4	188.2	107.67	0.25	0.18	N
PANI (30 wt. %)/Te nanorods [173]	298	102	102	105	0.21	0.16	N
PEDOT:PSS (20 wt. %)/Te nanorods [174]	298	~680	27.5	51.4	~0.16	~0.10 ^†^	PTh
PANI (70 wt. %)/Bi_2_Te_3_ [175]	300	~12	~36	~1.6	~0.11	~4.3 × 10^−3^	N
PANI/PbTe [176]	298	1.77	69	0.713	-	-	N
PEDOT:PSS( 5% DMSO)/Bi_2_Te_3_ [177]	298	945	22.2	47	-	-	PTh

**Table 8:** Thermoelectric properties of various oxide/polymer composites at a stated temperature (*T*). GINC = graphene–iron oxide nanocomposite. Gr = Graphene. Polyparaphenylene = PPP. *σ* is electrical conductivity, *S* is the Seebeck coefficient, PF is the power factor and κ is the thermal conductivity. All values are reported at *ZT*_max_, figure of merit at maximum or PF_max_ where applicable. ^†^ Calculated from literature values.

Composite	*T* (K)	σ	S (µV K^−1^)	PF (µW m^−1^ K^−2^)	κ	*ZT*	Polymer Class
p-type oxide/polymer composites							
PANI/BaTiO_3_ [188]	365	~270	~39	~41 ^†^	-	-	N
PEDOT/TiO_2_-ZnO [189]	383	~71	19.3	2.61	0.13	7.5 × 10^−3^	PTh
PANI (84 wt. %)/AlZnO [190]	298	~25	~54	~6.4	0.62	3.5 × 10^−3^	N
PANI (99.25 wt. %)/NiO [191]	383	~0.14	331	1.25	-	-	N
PEDOT:PSS (75 wt. %)/Fe_3_O_4_ [192]	300	~218	~16	~5.5	-	-	PTh
PEDOT:PSS (5 wt. %)/GINC [193]	300	~800	25.4	51.93	0.90	17 × 10^−3^	PTh
Matrimid (10 wt. %)/Ca_3_Co_4_O_9_ [194]	373	~0.2	~127	~0.32 ^†^	~1.16	~1.04 × 10^−4 †^	-
Matrimid (10 wt. %)/C (5 wt. %)/Ca_3_Co_4_O_9_ [194]	523	~1	~178	~3.17 ^†^	~0.49	~3.38 × 10^−3 †^	-
NiO/PPy/Gr [195]	373	1.15	495	28.22	-	-	NAr
n-type oxide/polymer composites							
PPP (0.95 wt. %)/Li_0.5_Ni_0.5_Fe_2_O_4_ [196]	820	~0.88 (800 K)	~−228	-	~1.86 (800 K)	0.11	-
PANI (80 wt. %)/SrTiO_3_ [197]	300	~49	~−100	~49.6	-	-	N

## Data Availability

Not applicable.
